# A study of location selection for large agricultural wholesale markets under the perspective of modern circulation

**DOI:** 10.1371/journal.pone.0345727

**Published:** 2026-04-07

**Authors:** Guizhe Xin, Yuqing Tang

**Affiliations:** 1 College of Architecture and Urban Planning, Tongji University, Shanghai, China; 2 Department of Design Management, Qingdao Conson Construction & Investment Co., Ltd., Qingdao, China; Southeast University, CHINA

## Abstract

This study investigates the critical challenges associated with location selection for large-scale Agricultural Product Wholesale Markets (APWMs) under the traditional circulation model. It identifies and elaborates on the evolving characteristics of circulation stakeholders, supply chains, distribution channels, organizational structures, and external environments during the transition from traditional to modern circulation systems. In response to the demands of modern circulation, a comprehensive location selection evaluation framework is proposed, integrating five key criteria: location, planning, transportation, land use, and urban compatibility. Elastic and rigid evaluation standards are established according to the nature of each criterion. The framework innovatively integrates national territorial and spatial planning, road traffic planning, industrial development planning, and urban big data resources through Geographic Information System (GIS) technology, consolidating these into a unified database. To determine comprehensive weights for different functional types of APWMs, the normalized linear aggregation method is applied to combine weights derived from the Analytic Hierarchy Process (AHP) and the entropy weight method, enabling an analysis of correlation and contribution levels. Furthermore, this study introduces an innovative application of the Genetic Algorithm (GA), implemented in Python, to re-optimize the integration of subjective and objective weights through iterative computation until convergence, thereby enhancing the accuracy of comprehensive weight estimation and validating the location selection outcomes. A case study demonstrates the successful development of a five-phase location selection methodology—region screening, scope delineation, data analysis, weight optimization, and comprehensive evaluation—enabling both quantitative ranking and recommendation of candidate location and the optimal solution was ultimately selected from seven candidate schemes. This research provides practical guidance for location selection of large-scale APWMs within modern circulation contexts and offers methodological insights applicable to urban logistics planning and the siting of other large-scale infrastructure facilities.

## Introduction

In China, the APWM is the primary channel for the circulation of agricultural products and occupies a central position in the overall system [1]. Statistical evidence indicates that over 80% of the city’s fresh products are sourced from this wholesale market [2]. The large-scale APWM functions as a critical element in the urban food supply chain, playing a pivotal role in various aspects such as price formation [3] circulation efficiency, food safety, agricultural industrialization, emergency protection [4], and more. The large-scale APWM generally refers to the first-level wholesale markets with an annual operation volume of 1 million tons or more up to over 3 million tons [5]. The 2023 No. 1 Central Document of China proposed the construction of a diversified food supply system and the establishment of a broad food perspective. Enhance the backbone network for agricultural product circulation by transforming and upgrading wholesale markets in production regions, distribution regions, and consumption regions, including: PWM, DWM and CWM. In 2024, the Ministry of Commerce, in conjunction with nine other departments of China, released the “Action Plan for Enhancing the Modern Commercial and Trade Circulation System and Promoting High-Quality Development of the Wholesale and Retail Sector.” The plan outlines the objective of establishing, by 2027, a modern commercial and trade circulation system that integrates domestic and international markets, connects urban and rural areas, links production with sales, and ensures efficient and smooth operations. Furthermore, the wholesale and retail sector is expected to accelerate its transformation, cultivate 100 key APWM, and continuously enhance its circulation organizational capabilities. The 2025 No. 1 Central Document of China proposes to expedite the development of a modern distribution network for agricultural products and agricultural supplies, while encouraging diverse entities to collaboratively construct supply chains. Establishing large-scale APWM is both urgent and highly significant for standardizing the agricultural product circulation system.

However, under the traditional circulation model, China’s agricultural products face several critical challenges: an excessive number of distribution stages, high logistics expenses, insufficient rates of cold chain storage and transportation, low levels of informatization and standardization, as well as imbalanced development attributed to a lack of modern trading platforms. The central issue lies in the location selection for APWM, which is associated with the following problems:

Inadequate location planning and insufficient comprehensive coverage: high-quality production regions, specialized cooperatives, and key consumption areas fail to establish full connectivity based on market attributes such as origin, sales, and distribution centers. This results in an increase in circulation costs.

Functional isolation and insufficient coordination: There is a lack of integration with relevant plans, such as territorial and spatial planning and industrial planning, to achieve mutual coordination with surrounding land functions. Additionally, the absence of corresponding specialized planning has resulted in disconnected links with the layout of urban warehousing, logistics, and distribution facilities, thereby trapping the extension of the industrial chain.

Inconvenient access and inefficient production-sales linkage: Many markets are situated far from main roads and deeply embedded within urban areas, leading to weak traffic diversion capabilities. Urban freight passage disrupts peace and tranquility. Cold chain storage and transportation connections are inadequate, hindering timely delivery. In China, post-harvest losses of agricultural products, particularly fresh vegetables and fruits, reach 20% to 30%, whereas in developed countries, these losses range from only 1% to 5% [6].

Dispersed layout and insufficient supporting infrastructure: The market is characterized by its fragmentation and limited size, with inadequate space for expansion. There is a paucity of facilities dedicated to trading, processing, inspection and quarantine, cold chain storage, logistics and distribution, research and development, and exhibition. This deficiency hinders the attainment of economies of scale and the facilitation of a shared economy.

Market-urban environment conflicts and mutual interference: Markets are increasingly surrounded by urban areas, exacerbating issues of a dirty, disorderly, and suboptimal trading environment [7]. These problems hinder the development and transformation of both the “market” and the “city,” necessitating urgent improvement and modernization.

Contemporary economic systems are characterised by the increasing volumes and flow of goods. The importance of geographical location is more prominent in the logistics process [8]. In location theory, location characteristics and efficiency are common focuses in planning and urban studies [9]. In location theory, under the influence of modern circulation, the connection between location and efficiency has become even closer. In addition to relatively efficient accessibility, the issue of fairness to service recipients in terms of location selection is also worthy of attention [10]. Just as the correct selection of the location for logistics centers is crucial for efficient trade and the economy [11]. The locational attributes of large-scale agricultural product wholesale markets are critical to the efficient functioning of the entire agricultural product distribution system.

Based on the aforementioned analysis, it is evident that whether constructing and nurturing new markets or relocating old ones, the scientific nature of location selection has emerged as the primary issue in resolving the contradiction between “market” and “city” and establishing a modern urban-rural agricultural product circulation system.

In 2023, the Chinese government released the “Notice on the Layout and Construction of Modern Circulation Strategic Pivot Cities,” designating 102 national modern circulation strategic pivot cities. The national “14th Five-Year Plan for the Construction of a Modern Circulation System” emphasizes accelerating the development of a modern circulation system, fostering innovative circulation organizations and business models, and promoting seamless integration across upstream and downstream sectors, production, supply, sales, and domestic and international trade. Modern circulation connects production with consumption through an extensive supply chain network and links supply and demand via more precise and efficient logistics and distribution systems. The circulation of agricultural products has transitioned from traditional models to modern ones, resulting in significant transformations in transactional relationships and logistics organization models [12–14]. This shift from traditional to modern circulation introduces numerous new characteristics and provides a novel framework for addressing locaion selection challenges under the traditional circulation paradigm.The research aims to develop a comprehensive locaion selection evaluation system in response to the development trends and demands of modern circulation. It focuses on selecting appropriate and operable evaluation methods, integrating them with territorial and spatial planning and industrial planning, to achieve quantitative assessments for the location selection of large-scale APWM including PWM, DWM and CWM. This study will support regions, such as China, which have an urgent need to establish APWM system, in designing effective location selection evaluation approaches and improving the efficiency of modern circulation systems.

## Literature review

### Modern circulation

In a previous study, Cadilhon et al. [15]. examined the efficacy of contemporary marketing channels in developing countries and their effect on traditional methods. Oparebea Boateng et al. [16] pointing out the tendency of supermarket purchasing to distribution centers, specialised/dedicated wholesalers, preferred supplier programmes in the context of the shift from traditional to modern distribution models. Xu [17] proposes a contemporary circulation model as a consequence of the modern logistics industry’s enhanced productivity. This model integrates “new logistics” and “new retail” in a symbiotic relationship, giving rise to innovative forms of logistics such as instant and agile, intelligent and autonomous logistics. Logistics is a manifestation of circulation, which has also undergone a major transformation under the influence of modern distribution trends. Danyluk [18] proposed logistics revolution has altered sociospatial processes at multiple sites along the supply chain. Characterized by a high degree flexibility and the dense interdigitation of the spaces of production and circulation. Cichosz [19] proposed determinants of innovative business models in the digital era Including Connectivity, Cooperation, Integration, Adaptiveness. In addition, Cichosz et al. [20] listed building information platforms, employee and partner engagement, and enhancing the customer experience for logistics companies under Digital transformation as the success factors for the transformation. The impact of modern circulation on the industry chain is also worthy of attention. Hu [21] proposed that the modern circulation industry should be integrated with related industries and innovation, vigorously developing the integration of urban and rural production, supply, and marketing of big data platforms, as well as the comprehensive integration of urban and rural production, supply, and marketing of various enterprises and farmers in the chain. Chen [22] proposed modern circulation is defined as a comprehensive, open, and dynamic process that encompasses the entire cycle of production, distribution, circulation, and consumption.

Modern circulation is embedded in urban mobility and is closely related to traffic dynamics, governance and policy integration mechanisms, and community equity [23]. When selecting locations for large-scale agricultural product wholesale markets, it is essential to comprehensively consider their roles within the circulation network and the various stakeholders associated with them. Additionally, leveraging information technology and efficient logistics infrastructure to establish interconnected systems can effectively enhance circulation efficiency.

### Location selection of wholesale market and logistics center

Large-scale APWM are important logistics, warehousing and distribution infrastructure. There have been numerous studies on the location selection of similar facilities such as logistics centers and wholesale markets. Aiming at the five aspects of the problems faced by traditional distribution in the previous study, the criteria influencing the location of wholesale markets or logistics centers for agricultural products listed in the literature were categorized and summarized in Table 1.

**Table 1:** Research status of location selection criteria.

Categorization	Criteria
**Regional location**	Xiao [24]: Distribution of stores, supply of production bases; Awasthi et al. [25]: Proximity to customers and suppliers; Agrebi and Abed [26]: Distance from suppliers; Dey et al. [27]: Proximity to customers, suppliers or producers; Yazdani et al. [28]: proximity to customers and suppliers; Uyanık et al. [29], Zhang [30]: Proximity to Center of City; Onstein et al. [31] Muerza et al. [32]: labour availability
**Functional association**	Ocampo et al. [33]: remanufactured product by the secondary market in an area; Muerza et al. [32]: Level of industry diversity, Closeness to logistics hubs; Özmen et al. [34]: Proximity to production centers, the scope of the city development plan; Dey et al. [27], Pham et al. [35]: Development policies for economic zone
**Transportation accessibility**	Yazdani et al. [28], Yu [36]: accessibility, connectivity to multimodal transport; Muerza et al. [32]: Availability and connectivity of expressway, capacity of railroads for freight transportation, intermodal transportation, Ease of access to seaport; Dey et al. [27], Zhang [30] Pham et al. [35]: Availability of railroads, expressways, seaport and airport.
**Land and facility supporting**	Uyanık et al. [29], Yu [36], Song [37]: Natural environment (meteorology, geology, topography, etc.); Muerza et al. [32] Infrastructure, Cost of land acquisition; Pham et al. [35], Awasthi et al. [25], Uyanık et al. [29]: Size, Capability for expansion of area.
**City friendly**	Awasthi et al. [25], Agrebi and Abed [26], Li [38], Song [37]: Environmental impact; Awasthi et al [25], Uyanık et al. [29]: Safety and Security; Kieu et al. [39]: Sustainability

For the method of selecting the location of agricultural markets or logistics centers, from the point of view of optimal logistical efficiency Özmen et al. [34], Xiao [24] selected key criteria were chosen for the locaion selection study；Raimbekov et al. [40] used AHP and centroid method, Muerza et al. [32] compared ANP algorithms, Kieu et al. [39] used hierarchical analysis (SF-AHP) and combined compromise solution (CoCoSo) algorithms, Pham et al. [35] used Delphi TOPSIS method, and the above studies were conducted at national and provincial regional level for locaion selection. Zhang [30] used entropy weight TOPSIS combined with GIS technology, Giuffrida et al. [41] used GIS combined with the Delphi method to conduct a study on the location of agricultural markets or logistics centers at the urban level. While for subjective or objective quantitative analysis has its own focus, Uyanık et al. [29] conducted a literature review of relevant locaion selection methods, and in China relevant normative standards, policy documents also do not have a detailed agreement or guidance on location selection evaluation methods.

Based on the aforementioned analysis, the location selection scope primarily focuses on urban areas, with an emphasis on municipal regions. The locaion selection methodology should not only encompass qualitative approaches but also integrate quantitative techniques by leveraging GIS and big data resources to analyze the spatial attributes of the city. The foundation for location selection must consolidate the outcomes of territorial and spatial planning and industrial planning, ensuring a forward-looking and operational framework. More importantly, it is essential to integrate the features of modern circulation by forming a comprehensive distribution chain. Through a combined subjective and objective analytical approach, full-criteria quantification should be conducted to enhance the scientific rigor of locaion selection research for markets with varying functional attributes.

#### Urban planning and accessibility impacts.

Large-scale APWMs play a significant role in urban planning and, in turn, exert a substantial influence on urban development strategies. Charters-Gabanek et al. [42] review presents a broad discussion of the links between the logistics industry and a series of urban planning concerns, develop a framework around the forces shaping the supply of, and demand for, land for logistics to engage with current conversations in urban studies on the production of urban spaces. Kin et al. [43] put forward cities can support the (re)integration of logistics facilities in urban areas to facilitate and enable the shift to an efficient urban logistics system. Develop best practices on how to address the integration of urban logistics facilities for cities. Wang et al. [44] use the real data of Beijing analysis of the accessibility impacts of logistics suburbanization, highlights the potential for freight accessibility in urban freight planning. Chen et al. [45] By leveraging existing urban infrastructure, a multi-criteria evaluation framework integrating the AHP and the TOPSIS was developed to support the location selection of urban logistics centers. Furthermore, in the research on the impact of transportation accessibility on vulnerable groups, Abdulrazaq et al. [46] established a priority evaluation framework for vulnerable road users and developed a spatio-temporal cube analysis approach by integrating XGBoost to assess the impact of emerging factors on traffic accessibility.

## Methodology

### Location selection strategy for modern circulation

Through a comprehensive review of modern circulation-related literature and an analysis of relevant policies, it is evident that the development of agricultural product wholesale markets must align with the requirements of modern circulation systems. This involves integrating multi-level resources to create favorable conditions for establishing a modern circulation platform that encompasses production, supply, sales, processing, cold chains, warehousing, distribution, and information interconnectivity. Based on the five key issues inherent in traditional circulation models, this study specifically examines the new characteristics exhibited by circulation entities, circulation chains, circulation channels, circulation organizations, and circulation environments during the transition from traditional to modern circulation. Additionally, problem-oriented considerations for the location selection of large-scale agricultural product wholesale markets are systematically outlined in Table 2.

**Table 2:** New transformation and location selection orientation.

Shifting trends	Form of expression	Location orientation
**Circulation subject shifted from limited to extensive**	In the context of contemporary trends in digitalization, sharing, experientialization, chaining, disintermediation, express delivery, and the separation of commercial flow from logistics [47], various types of wholesalers, producers, sellers, and logistics companies are engaged in the process of distribution chain formation. The objective of this collaboration is to facilitate comprehensive cross-domain resource integration.	Integrate the circulation subjects; Implement location synergy
**Circulation chain shifted from fragmentation to interconnection**	The efficiency of the link between production, supply, and marketing has become paramount in modern circulation. In response to consumer demand, the supply chain ecosystem is subject to constant adjustments, thus forming a closed loop that encompasses production, distribution, circulation, and consumption [22].	Connecting Independent Functions; Interconnecting layouts
**Distribution channels shifted from single to multidimensional**	The advent of the Internet and big data technology has led to a more precise and efficient matching of supply and demand, resulting in a multifaceted logistics network that integrates online and offline, terrestrial, maritime, and aerial transportation methods.	Expand distribution channels; Improve accessibility
**Circulation organization shifted from decentralized to integrated**	In contrast, at the core of the industrial chain, characterized by its “low, small and scattered” nature [48], there is a steady concentration of resources within channels that exhibit higher levels of circulation efficiency. Large-scale circulation enterprises emerge as dominant entities, orchestrating the integration of services within the industrial, supply, service and innovation chains [49].	Integration of distribution services; Optimizing system organization
**Circulation environment shifted from chaos to order**	The orderly construction of the national unified market, modern circulation tends to production, supply and marketing intelligent linkage, service customization, environmental friendliness, process visibility and other characteristics, in the face of emergencies to show a stronger resilience and emergency protection capacity [50].	Solve the contradiction between the city and the market; Regulating the supply

### The establishment of the location selection evaluation system for new circulation

In light of the aforementioned five major issues in traditional circulation and the transition from the five characteristics of traditional circulation to modern circulation, the location orientation is transformed into an evaluable influencing criterion. By integrating existing research, location criteria are consolidated, supplemented, refined, or quantified. Consequently, a location evaluation system comprising five primary influencing criteria is constructed to fulfill the requirements of modern circulation.

#### Location synergy (L) -- integration of multiple market systems.

**Fig 1:**
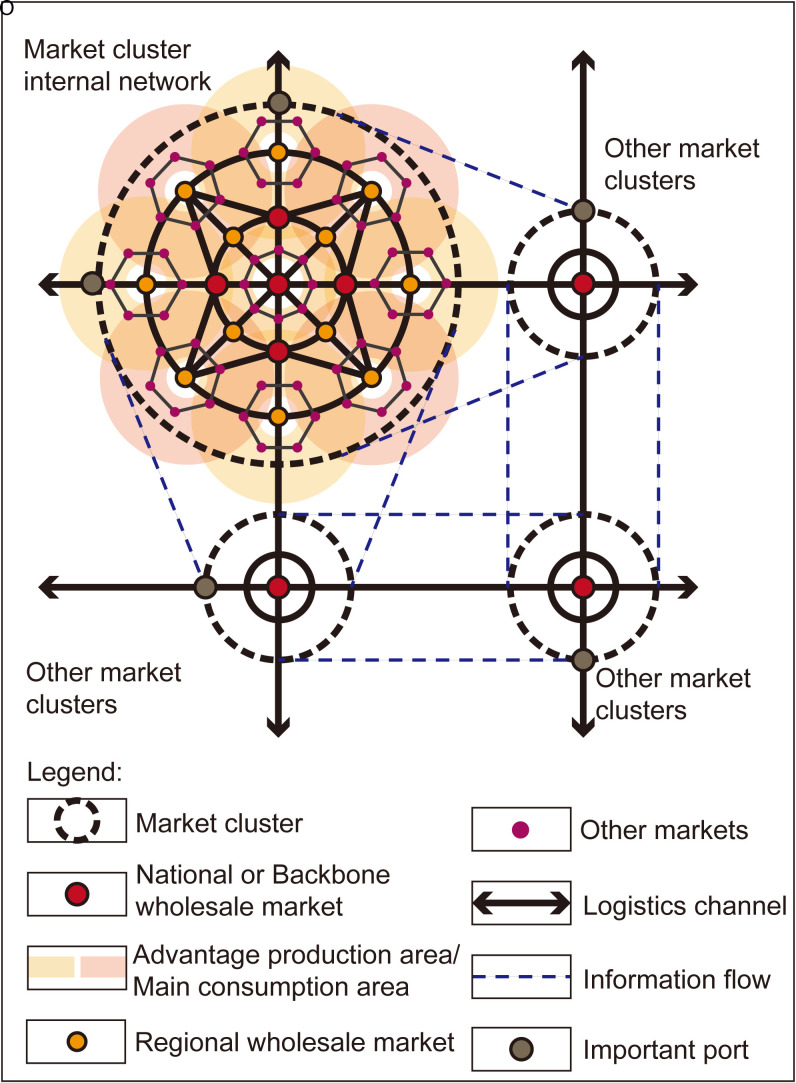
Macroscopic circulation network of agricultural products market. Caption credit: This figure was created by the authors using Adobe Illustrator.

In light of the location coordination issue, existing studies have investigated the correlation between production and consumption locations [25,26,34] as well as the allocation of resources in central urban areas [29,30,34]. Within the framework of modern circulation systems, market selection should emphasize hierarchical refinement and the linkage relationships among markets at various levels. This involves integrating the “three vertical and three horizontal” backbone network for national agricultural product circulation [51] with a three-tiered agricultural product origin market system [7], which is structured with the national level as the leader, regional level as the node, and field markets as the foundation. Consequently, this forms a circulation pattern characterized by orderly linkage, with national or backbone markets and regional markets serving as primary nodes as show in (Fig 1).

**Fig 2:**
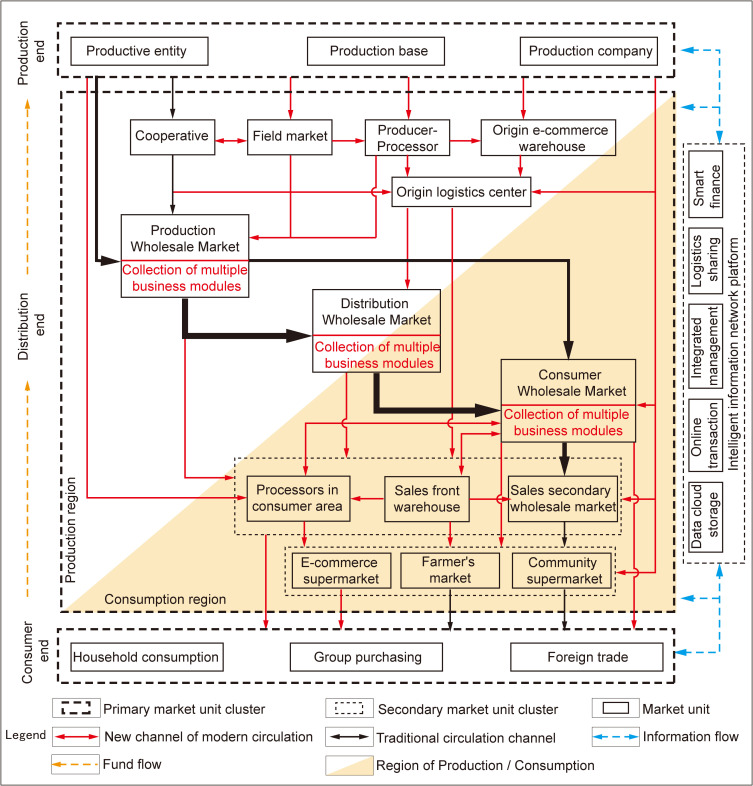
Simulation of the circulation process of agricultural products under the modern circulation model. Collection of multiple business modules include: E-commerce, cold chain, Storage, processing, distribution module. Caption credit: This figure was created by the authors using Adobe Illustrator.

At the micro level, modern circulation relies on multi-channel interactions from the production end to the consumption end, guided by an overarching smart platform. This has given rise to numerous modern circulation pathways as show in (Fig 2), such as “agricultural wholesale and retail connection,” “agricultural supermarket connection,” “agricultural community connection,” and “order-based agriculture.”

On the one hand, the regional layout is well-coordinated (L1). By considering the distinct functional attributes of production, consumption, and distribution markets, the market entity is further categorized into: external city markets (L1-1), advantageous production areas within the city (L1-2), lower-level origin market (L1-3), lower-level consumption markets (L1-4), farmers’ markets (L1-5), and major supermarkets (L1-6). This classification ensures comprehensive coverage of connections with various market entities and facilitates a comparative analysis of circulation costs. On the other hand, in light of the integration of modern circulation with human resources, market clusters, and public service facilities within the central urban area, and adhering to compliance requirements such as “Planning for the One-Hour Fresh Agricultural Products Circulation Circle around the Capital” and “Management Specifications for Instant Retail Operations (DB42/T 2325-2024),” attention is given to the one-hour delivery radius in relation to the central urban area (L2), which serves as a rigid criterion for locaion selection.

#### Planning coordination (P) – Interconnecting multi-functional spaces.

For the functional planning problem, studies have primarily focused on “proximity to organized industrial zones,” the scope of urban development in terms of “urban development planning” [34], and industrial policy [7]. Modern circulation emphasizes the establishment and expansion of a complete industrial chain. Locaion selection should integrate the requirements of various levels of territorial and spatial planning and industrial planning regarding the layout of industrial functional spaces, thereby enabling comprehensive overall planning and constructing an integrated network.

**Fig 3:**
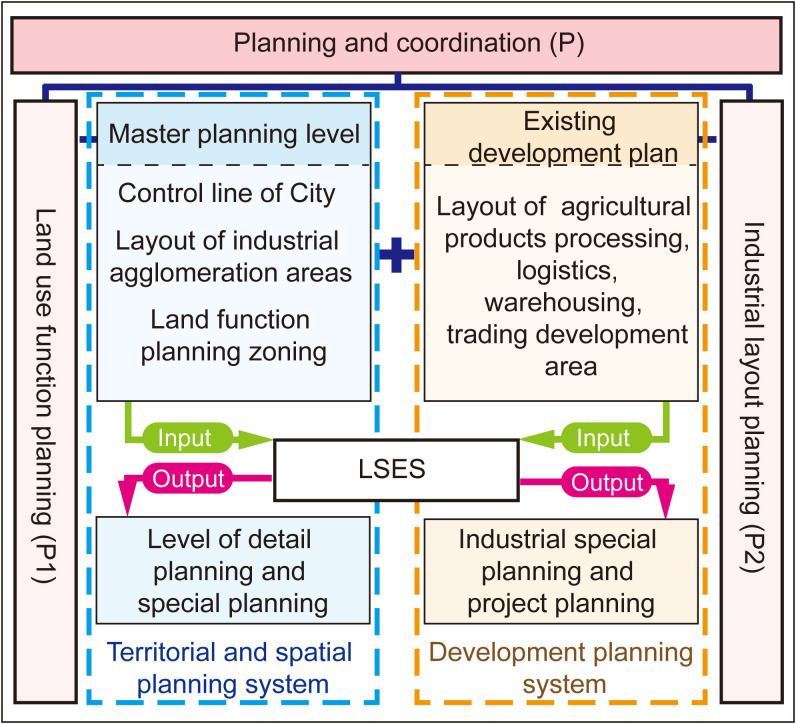
Influence relationship between location selection evaluation and planning. LSES, Location selection evaluation system. Caption credit: This figure was created by the authors using Adobe Illustrator 2023.

Link up the territorial and spatial planning system and coordinate the functional layout of land use (P1): To align with the content and requirements of the comprehensive territorial and spatial planning, location selection must consider its dual role as both a transit station for agricultural products outside the city area and a supply and security station for urban agricultural products. Based on spatial-temporal evolution research, such locations are predominantly distributed in inner or outer urban areas [52]. This guides location selection to adhere to the suburbanization principle of “close to the city but not within it,” as exemplified by cities listed in Table 3. A rigid principle for location selection is that the location must be situated near or within the urban development boundary. Regarding land use, wholesale market land falls under commercial land according to land use standard (Guide to classification of land and sea use for territorial space survey, planning and use control) in China. In conjunction with existing land function plans, it is imperative not to occupy residential, public service facilities, special-purpose land, scenic spots, or other designated areas as a fundamental planning principle. Looking ahead, considering the integration of transaction, incubation, processing, and logistics industry chains for large-scale agricultural product wholesale markets under modern circulation systems, priority should be given to locating industrial land such as commercial service, storage, and industrial land. Additionally, by combining industrial control line demarcations, extract big data resources such as POI related to agricultural products or food processing, logistics warehousing, and cold chain enterprises. Select relatively clustered industrial development zones, compare the correlation degree of candidate sites, and incorporate elastic evaluation indicators. In the later stages, locaion selection results will be validated and adjusted through detailed or specialized planning as show in (Fig 3).

**Table 3:** Planning and layout of large-scale APWM in some big cities in China.

City	Planning and layout	Distance from city center (km)	Related planning
**Beijing**	There are four comprehensive first-level wholesale markets planned in the southwest, southeast, northeast and northwest of the main city.	15-20	Development Plan of Agricultural Products Circulation System in Beijing during the 14th Five-Year Plan Period
**Shanghai**	“4 + 6+N”: The construction of four major wholesale markets in the city from the southeast to the northwest	13-38	Shanghai Commercial Space Layout Special Plan (2022–2035)
**Chongqing**	“1 + 1+16”: the main city metropolitan area to upgrade a national influence of the cross-regional APWM, the cultivation and construction of a cross-regional distribution and distribution of APWM	30-65	Planning and Layout of Wholesale Agricultural Products Market in Chongqing Metropolitan Area (2021–2025)

Adapted from: related planning listed in “Related Planning”.

Integrate the industrial spatial layout (P2): Link existing plans, and consolidate industrial development strategies with specialized plans such as agricultural modernization, food industrialization, cold chain storage and distribution, and logistics development. Evaluate the correlation of relevant industrial parks, processing parks, logistics parks, and other key industrial development zones as an elastic evaluation criterion for locaion selection. Simultaneously, transmit the outcomes of the location selection process to industrial special planning and project planning as show in (Fig 3).

#### Transportation accessibility (T) -- Expanding multi-dimensional circulation channels.

In addressing the issue of transportation connectivity, modern agricultural product circulation relies on efficient information interconnection to rapidly establish logistics connections among production, supply, and sales. Concurrently, various e-commerce models have emerged, such as “fresh food e-commerce + cold chain home delivery,” “central kitchen + cold chain food delivery,” O2O (online-to-offline), and C2P (consumer-to-producer) [14]. Modern circulation tends to customize circulation plans for different channels, select optimal circulation paths as shown in (Fig 2), and facilitate access to multi-level transportation networks both domestically and internationally. In conjunction with existing research on traffic impact [29,30] five key traffic impact factors are established: intercity expressways (T1), urban transportation (T2), freight railway facilities (T3), port and wharf facilities (T4), and airport facilities (T5). Emphasis is placed on the seamless integration of intermodal transport and personalized transportation demands within modern circulation systems. A comprehensive quantitative analysis is conducted to balance the rigidity and elasticity of all criteria involved.

Given that over 75% of agricultural products are transported via road networks [40], where expressway accessibility is a critical factor influencing the efficiency of commodity logistics [53]. Efforts should be made to minimize the distance between markets and expressways, enhance operational efficiency, and reduce urban impacts. Drawing from investigations of major agricultural markets, such as Rungis in France, Toyosu market in Japan, and Xinfadi market in Beijing, a maximum traffic connection time of 5 minutes to expressway entrances and exits is recommended.

Considering the increasing proportion of air transport for agricultural products, the low-altitude economy has established a modern circulation pathway for agricultural product transportation. Air cargo not only enhances the value of transported goods but also broadens the range of circulation participants [54]. This highlights the importance of connecting locaion selection with various levels of airports. According to research findings, logistics facilities strongly associated with airports are typically located within 20 kilometers of an airport [55,56]. A driving limit of 30 minutes from the airport is defined based on average freight speed of 40 km/h [57]. and the control time for minimizing conventional transportation losses of fresh goods entering and leaving the port. The “urban transport (T2)” aspect considers its significant circulation and traffic demands, emphasizing direct connections with urban trunk roads or urban expressway (T2-1). These three points serve as rigid principles. Additionally, in response to the accessibility needs of diverse groups in the agricultural batch market under modern circulation, public transport accessibility (T2-2) evaluation indicators are introduced to comprehensively assess the overall transportation conditions of the selected location.

#### Land suitability (S) – Supporting the whole chain circulation service.

In response to the layout of supporting issues, the agricultural product wholesale market within the modern circulation system tends to establish a large-scale intelligent circulation platform that integrates the entire industry chain, including live e-commerce, processing and warehousing, cold chain logistics, centralized quarantine, expos, research and development incubation, recycling and reuse. This platform aims to provide comprehensive, one-stop, and highly efficient services. To ensure the successful implementation of such a platform, several critical criteria must be considered: sufficient scale and expandability (S1), strong industrial foundation (S2), reasonable land costs (S3), favorable land consolidation conditions (S4), and an appropriate environment (S5), including suitable construction site conditions (S5-1) and landscape features that facilitate market integration into the city (F5-2). Among these criteria, “Land use scale and expandability (S1)” is derived from domestic large-scale agricultural market research, referencing the size of urban-level wholesale markets exceeding 66.67 hectares [58], as well as the construction area and floor area ratio standards for large-scale APWM with an annual transaction volume of 300 tons or more [5]. Based on this, a recommended rigid locaion selection value of at least 65 hectares is established. Integrate the planning of suitable construction areas, as determined by the “two evaluation” of territorial and spatial planning, into the section “Site Conditions (S5-1).” In compliance with normative requirements (Vertical Planning Code for Urban and Rural Construction Land (CJJ83−2016) published by China), strive to ensure that the natural slope of the land does not exceed 15% wherever possible, thereby minimizing construction impacts resulting from adverse conditions such as natural disasters. Considering the modernization of the business format in APWM and the need to enhance the quality of the construction environment, integrate with the territorial and spatial planning system. The planned park green space within an 800-meter buffer zone that can be reached by walking at a comfortable adult walking speed of approximately 0.9 m/s [59] is correlated with the “landscape conditions (S5-2)” and integrated into the locaion selection evaluation framework. This promotes the transformation of the market into a new driver for characteristic cultural tourism and urban vitality enhancement.

#### City friendly (F) – Standardize the operational sequence of multiple types.

In response to the issue of urban interference, modern circulation methods have become increasingly diverse and adaptable. These methods not only ensure timely delivery to downstream markets or direct consumers but also contribute to maintaining a peaceful and orderly urban environment. To achieve modern circulation objectives, it is necessary to introduce concepts and standardized guidelines that facilitate quantifiable and expanded evaluation indicators. Drawing on the principles outlined in the “Technical Guide for Community Living Circle Planning” and the “Guide for the Construction of a Quarter-Hour Convenient Living Circle in Cities,” part of the APWM could be transformed into a 15-minute convenient distribution system. This system would enable prompt distribution while avoiding homogeneous competition. Additionally, a rigid principle should be established, ensuring a minimum distance of more than 15 minutes between existing markets (F1-1). Such measures aim to establish a regionally responsive, flexible, and convenient supply system. Under modern circulation, it is essential to emphasize the integration of epidemic prevention with peacetime and emergency preparedness, ensuring the smooth operation of the emergency support system. Additionally, the evaluation of the efficiency of urban logistics and emergency support facilities (F1-2) should be enhanced, and the agricultural product circulation system should be incorporated into the framework of urban safety and resilience guarantees. To foster a peaceful and orderly urban living environment while preventing pollution, it is recommended that the distance between residential areas and large public service facilities such as education and healthcare (F2-1), as well as toxic and hazardous pollution sources or dangerous sites (F2-2), be maintained at more than 1 km [60,61] as a rigid condition. This ensures minimal interference and impact on residential areas and large public service facilities, while simultaneously safeguarding the operational environment of agricultural markets. These measures collectively reflect the goal of fostering city-city amity under the context of modern circulation.

To ensure alignment between policies and established standards, Table 4 outlines the correspondence among policies and related research findings across multiple regions, thereby facilitating a clearer understanding of the relationship between the evaluation criteria adopted in this study and existing scholarly work.

**Table 4:** The relationship between the policies and criteria referenced.

Primary criteria (code)	Content of policy	Source of policy
**Location synergy (L)**	[7]	Ministry of Agriculture and Rural Affairs of the People’s Republic of China
	[51]	Ministry of Commerce of the People’s Republic of China
	Planning for the One-Hour Fresh Agricultural Products Circulation Circle around the Capital	Beijing-Tianjin-Hebei Region, China
	Management Specifications for Instant Retail Operations (DB42/T 2325−2024);	Hubei Province, China
	Planning and Layout of Large-scale APWM in Some Big Cities in China	Beijing, Shanghai, Chongqing, of China
**Planning coordination (P)**	[7]	Ministry of Agriculture and Rural Affairs of the People’s Republic of China
	Guide to classification of land and sea use for territorial space survey, planning and use control	Ministry of Natural Resources of the People’s Republic of China
**Transportation -accessibility (T)**	Case Study on High-Speed Accessibility	Rungis in France, Toyosu market in Japan, and Xinfadi market in Beijing
	Cases of the Association between Airports and Logistics Facilities [55,56].	Amazon fulfilment facilities in the United Kingdom and Japan；Airports access evidence from China
**Land suitability (S)**	[58]	Chongqing Municipal People’s Government of China
	[5]	All China Federation of Supply and Marketing
	Vertical Planning Code for Urban and Rural Construction Land (CJJ83–2016)	Ministry of Housing and Urban-Rural Development of the People’s Republic of China
**City friendly (F)**	Technical Guide for Community Living Circle Planning (TD/T 1062–2021)	Ministry of Natural Resources of the People’s Republic of China
	Guide for the Construction of a Quarter-Hour Convenient Living Circle in Cities	Ministry of Commerce of the People’s Republic of China
	[60]	Ministry of Housing and Urban-Rural Development of the People’s Republic of China
	[61]	Qingdao Market Supervision and Administration Bureau

To summarize, a location selection evaluation system has been established, comprising 5 primary location selection criteria, 16 secondary location selection criteria, and 25 sub-classification criteria. The overall criteria classification, along with other elastic evaluation principles, is elaborated in Table 5. Transportation convenience and cost are important criteria to be considered in location selection [29], while time cost often has a greater impact on logistics efficiency [62]. Therefore, in order to ensure fairness, the ADT to one or more analytical destinations is adopted as a key metric in specific elements for comparing circulation benefits.

**Table 5:** Evaluation table for location selection criteria in new circulation perspective.

**Location selection criteria**			**Evaluation principles**			**Units**
**Primary criteria(code)**	**Secondary criteria code**	**Secondary criteria**	**Principles of elasticity (including sub-classification criteria)**	**Principle of rigidity**	**Beneficial/ Cost**	
**Location synergy (L)**	L1	Link production and consumption	L1-1: ADT with external city markets	—	C	min
			L1-2: ADT with the city’s advantageous production areas			
			L1-3: ADT with lower-level origin market			
			L1-4: ADT with lower-level consumption markets			
			L1-5: ADT with farmers markets			
			L1-6: ADT with major supermarket			
	L2	Distance from central city	Driving time	t ≤ 60	C	min
**Planning coordination (P)**	P1	Land use function layout planning	ADT within the area delineated by the industrial control line, where major agricultural product processing, logistics, and warehousing industries are concentrated	Within the urban development boundary and as close as possible to the urban development boundary, do not occupy residential, public service facilities, special land, scenic spot land	C	min
	P2	Industrial layout planning	ADT of agricultural products processing, logistics, warehousing, trading industry development area	—	C	min
**Transportation accessibility (T)**	T1	Intercity expressway	The driving time to the nearest expressway entrance and exit	t ≤ 5	C	min
	T2	Urban transportation	T2-1: The length along the urban trunk roads or urban expressway	Located along a urban trunk road or urban expressway	B	m
			T2-2: Public transport accessibility: The average commuting time of public transportation at urban centers and arge passenger transport hubs	—	C	min
	T3	Freight rail facilities	ADT with the railway freight station	—	C	min
	T4	Port and wharf facilities	ADT with major port and wharf facilities	—	C	min
	T5	Airport facilities	Driving time with nearest airport facility	t ≤ 30	C	min
**Land suitability (S)**	S1	Land use scale and expandability	The extent of the surrounding expandable land that aligns with the territorial and spatial planning	S ≥ 65 ha	B	ha
	S2	Industrial background	The scale of land used for warehousing, logistics and food processing industries within 1 km of the location selection	—	B	ha
	S3	Cost of land	Regional land price	—	C	yuan/㎡
	S4	Land consolidation	Ground-level attachments’ floor area	—	C	㎡
	S5	Environmental suitability	S5-1: Site condition: the natural slope of the site is < 15%.	within the “Two Evaluation” suitable construction area of the territorial and spatial planning	B	%
			S5-2: Landscape conditions: The planned area of park green space within a 0.8 km range.	—	B	ha
**City friendly (F)**	F1	Balanced layout for emergency supply guarantee	F1-1: Interval for Supply Guarantee Based on Current Market Zoning: The driving travel time to the nearest large-scale APWM	t ≥ 15	B	min
			F1-2: Contact safety emergency facilities: ADT to the logistics support center and the emergency rescue material storage facility	—	C	min
	F2	NIMBY residential public service facilities and hazard sources	F2-1: NIMBY residential clusters or large public service facilities: Nimby distance	d ≥ 1000	B	m
			F2-2: NIMBY-related pollution sources include distances from waste treatment facilities, sewage treatment plants, thermal power stations, polluting enterprises, and hazardous sites (such as warehouses storing flammable, explosive, or other dangerous materials).	d ≥ 1000	B	m

Two Evaluation, Evaluation of the carrying capacity of resources and environment, as well as the suitability of territorial and spatial development.

### Evaluation methodology

Based on the integration of rigid and elastic approaches within the hierarchical framework of the location selection evaluation system in Table 5, various location selection methodologies are systematically categorized. Among these, the AHP has emerged as one of the most widely adopted techniques for logistics location selection [29]. AHP facilitates the organization of expert input from diverse fields, enabling the establishment of a layered evaluation matrix that aligns with the multi-level assessment system for location selection criteria.

Additionally, the entropy weight method provides an objective approach to weight calculation by leveraging data sourced from GIS, big data platforms, and other relevant sources. Consequently, the combination of AHP and the entropy weight method is employed for location selection evaluation. This integrated approach not only ensures the accurate computation of weights based on objective data but also accommodates expert adjustments to criterion weights in accordance with the specific requirements of different cities and markets.

**Fig 4:**
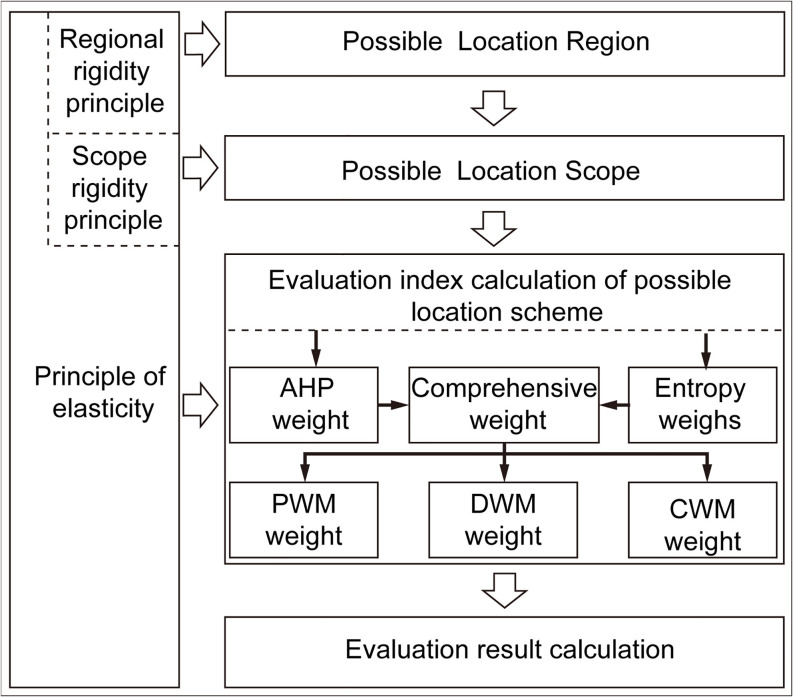
Operation mechanism of location selection evaluation. Caption credit: This figure was created by the authors using Adobe Illustrator 2023.

According to the classification and evaluation criteria presented in Table 5, the location selection evaluation system is implemented through a structured process that includes region selection, scope definition, weight determination, data analysis, and comprehensive evaluation. This process facilitates the location selection assessment for different types of large-scale APWM, including PWM, DWM, and CWM, as illustrated in (Fig 4).

## Data and results – the case of Qingdao city

### Establishment of location selection data base

Qingdao is situated in the Huang-Huai-Hai Plain National Quality Agricultural Production Area and serves as an international comprehensive transportation hub city, strategically positioned within the “three vertical and three horizontal” agricultural products circulation backbone network, which is also a modern circulation pivot city determined by the state. Based on the location selection criteria and principles outlined in Table 5, take the municipal boundary of Qingdao as the research scope, this study integrates the Territorial Spatial Planning of Qingdao (2021–2035), the Comprehensive Transportation System Plan of Qingdao (2021–2035), the territorial spatial planning of other districts in Qingdao City, relevant 14th Five-Year Plans, and other specialized plans of Qingdao City. Open-source data such as Open Street Map (OSM), DEM data, and big data POI are extracted to establish the necessary data resources for evaluation. The speed limit requirement of 100 km/h for freight vehicles on intercity expressways in China, combined with the statistics provided by the Bureau of Transportation Statistics of the U.S Department of Transportation, the average speed of trucks on interstate highways in 2019 ranged approximately from 48 to 62 miles per hour [53,63] (77–100 kilometers per hour), take the average value 90 km/h as the simulated average driving speed of the intercity expressway. Urban expressway, trunk roads, and secondary trunk roads, given the traffic congestion typically experienced in urban areas, reference should be made to the “Code for Design of Urban Road Engineering (CJJ37-2012),” adopting the minimum design speed as specified, set the average driving speeds of 60 km/h, 40 km/h, and 30 km/h.

**Fig 5:**
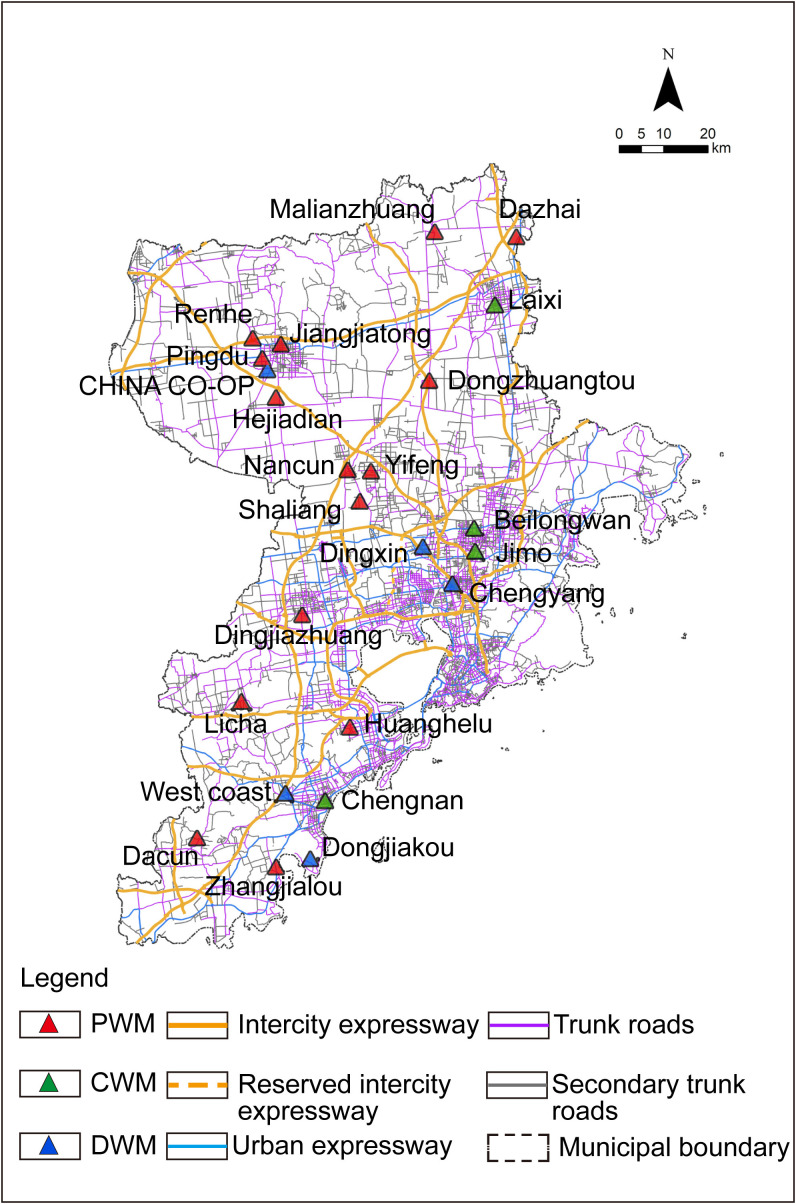
Location selection database of Qingdao. Caption credit: This figure was created by the authors using ArcGIS 10.8.1, Adobe Illustrator 2023.

As for the location data of the existing market, it mainly comes from the Qingdao Municipal Bureau of Commerce, the Qingdao Municipal Market Supervision Administration commissioned the Qingdao Municipal Urban Planning and Design Institute to compile the Qingdao Municipal Commercial Network Special Plan (2020–2035) and the Qingdao Agricultural Market Special Plan (2021–2035) respectively. According to the official website of the market and the above plan, the three types of large-scale APWM including PWM, CWM, and DWM within the city are identified as base markets and incorporated into the established database as show in (Fig 5).

### Region selection analysis

**Fig 6:**
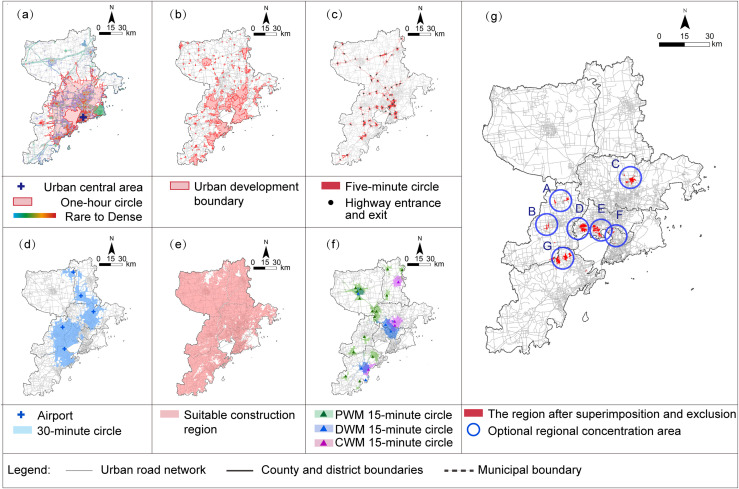
Analysis of Location selection region under the rigid principle. (a) L2: One-hour isochrone distance from the central urban area; (b) P1: Analysis of Urban Development Boundaries; (c) T1: 5-minute isochrone distance from entrance and exit of expressways; (d) T5: 30-minute isochrone distance from airport; (e) S5-1: Analysis of Suitable construction region; (f) F1-1: 15-minute isochrone distance from current different types of wholesale markets; (g) Analysis results of superimposed exclusion under the rigid principle of six criteria. Caption credit: This figure was created by the authors using ArcGIS 10.8.1, Adobe Illustrator 2023.

The stringent conditions associated with L2, P1, T1, T5, S5, and F1-1 were computed using GIS. Specifically, vehicle travel isochrone service area calculations were performed for L2, T1, T5, and F1-1. The results of L2, P1, T1, T5, and S5 were aggregated, while the analysis range of F1-1 was excluded. This process identified a total of seven potential location selection areas: A, B, C, D, E, F, and G as shown in (Fig 6).

**Fig 7:**
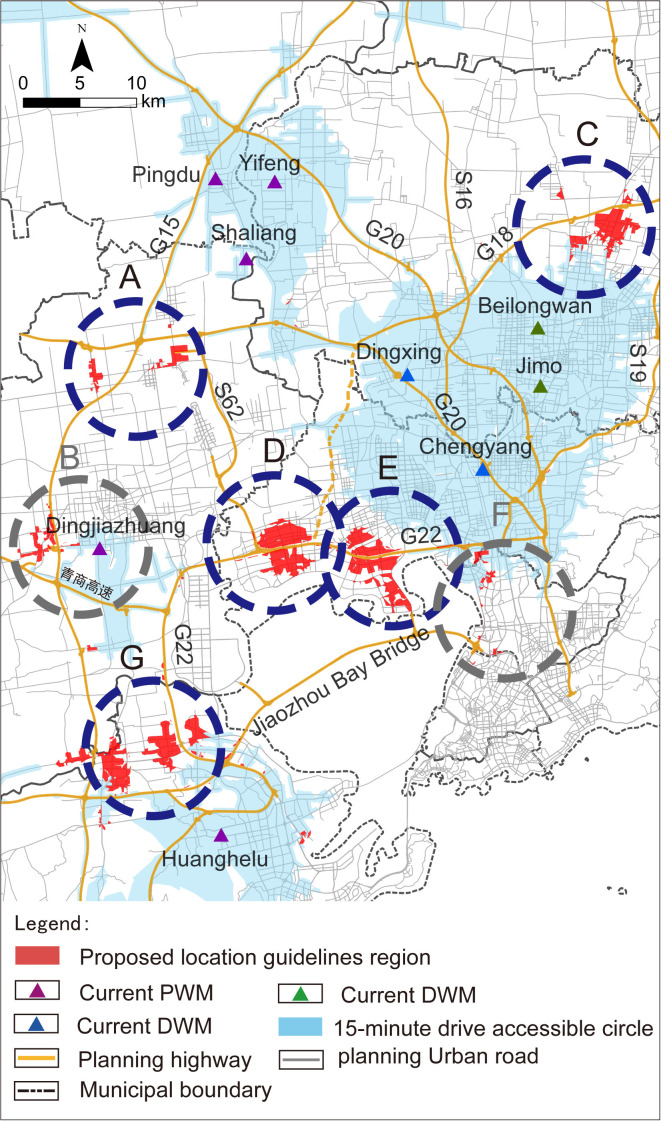
Analysis results of the proposed location selection region. Caption credit: This figure was created by the authors using ArcGIS 10.8.1, Adobe Illustrator 2023.

Subsequently, adjacent areas within the 15-minute isochrone of the existing market (F1-1) were disregarded, areas B and F are excluded, resulting in the final screening of five areas: A, C, D, E, and G as shown in (Fig 7).

### Scope definition analysis

**Fig 8:**
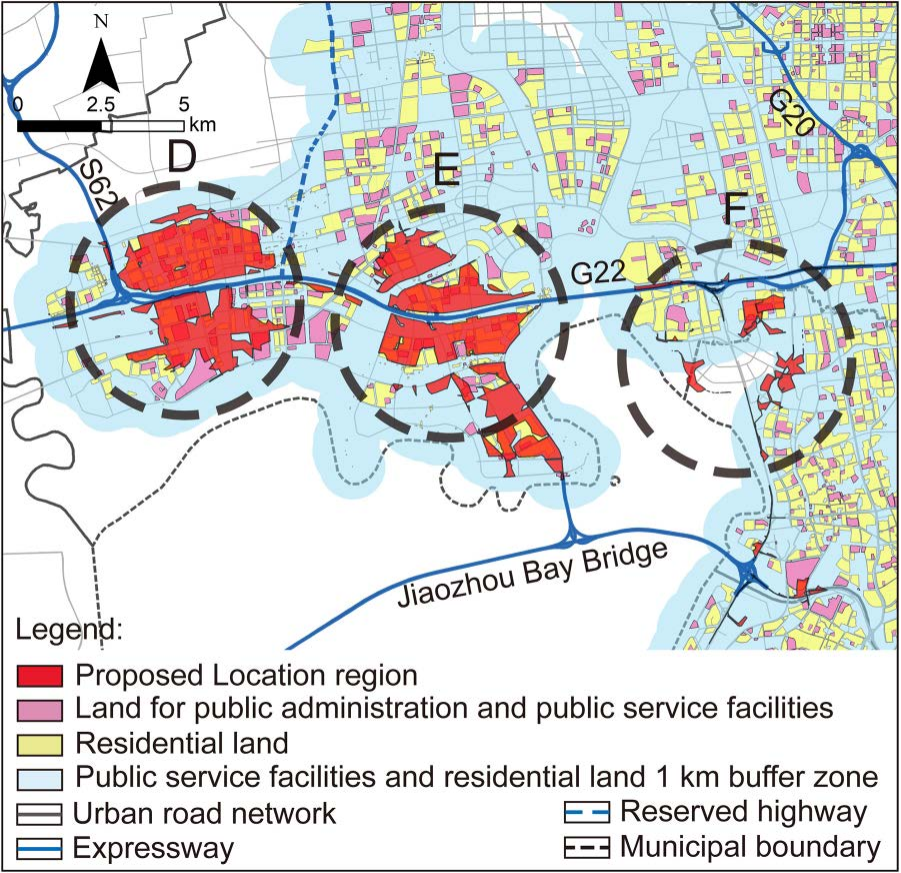
NIMBY relationship between region and residential and public service facilities. Caption credit: This figure was created by the authors using ArcGIS 10.8.1, Adobe Illustrator 2023.

**Fig 9:**
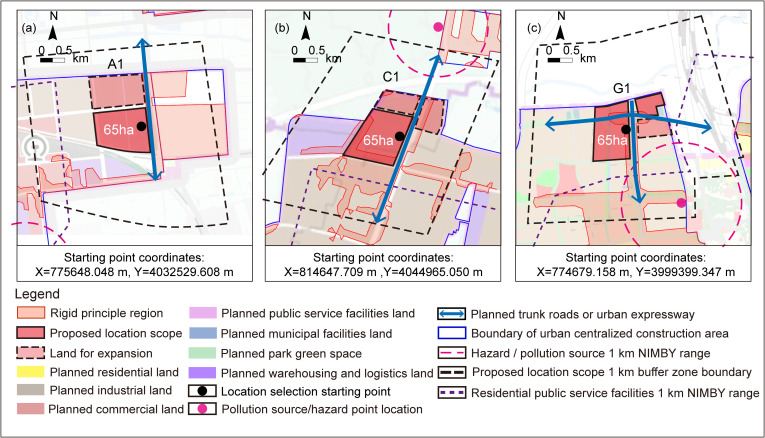
Analysis of the location scope for regions A1, C1, and G1 under rigid principles analysis. (a) Analysis of the surrounding region of location A1; (b) Analysis of the surrounding region of location C1; (c) Analysis of the surrounding region of location G1. The coordinate system uses the CGCS2000 (Gauss-Kruger 3° Zone 40) coordinates, where X represents northward coordinate and Y represents eastward coordinate, including zone number. Caption credit: This figure was created by the authors using ArcGIS 10.8.1, Adobe Illustrator 2023.

For the areas encompassed within or adjacent to the proposed location selection scope, the stringent criteria associated with operational criteria T2, S1, and F2 were applied to screen the proposed location selection scope. Following this process, areas D, E, and F were excluded from the location selection due to NIMBY concerns related to residential and public service facilities in (Fig 8). Since area F has already been excluded in the “Region selection analysis”, therefore areas A, C, and G each identified one suitable location selection scope, as outlined in (Fig 9).

### The calculation of the elastic principle criteria

With reference to 13 relevant plans and big data resources from Qingdao City, data computation or statistical analysis was performed for the evaluation indicators of the three proposed location selection scope.

**Fig 10:**
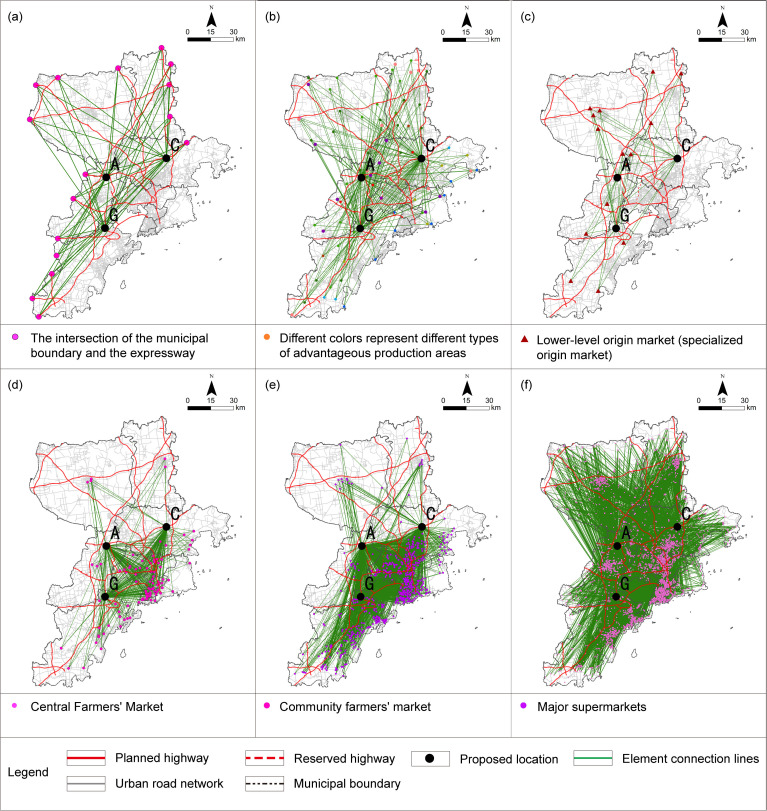
The analysis process of evaluation criteria from L1-1 to L1-6 under the elastic principle. (a) L1-1: ADT at the intersection where expressways meet the municipal boundary; (b) L1-2: ADT with the advantageous production areas; (c) L1-3: ADT with the secondary production market; (d) L1-4: ADT with the central farmers’ market; (e) L1-5: ADT with the community farmers’ market; (f) L1-6: ADT with the major supermarket. Caption credit: This figure was created by the authors using ArcGIS 10.8.1, Adobe Illustrator 2023.

**Fig 11:**
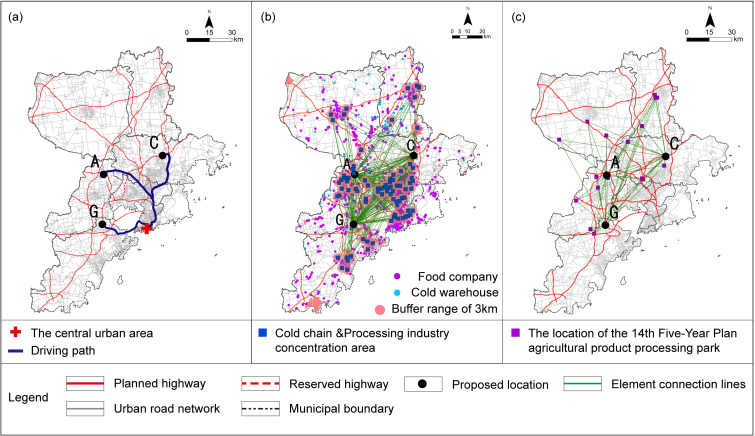
The analysis process of evaluation criteria from L2, P1 and P2 under the elastic principle. (a) L2: The shortest driving time to the city center; (b) P1: ADT of the food processing and cold chain logistics industrial concentration region in the territorial and spatial planning; (c) P2: ADT with the agricultural product processing park planned for the 14th Five-Year Plan. Caption credit: This figure was created by the authors using ArcGIS 10.8.1, Adobe Illustrator 2023.

**Fig 12:**
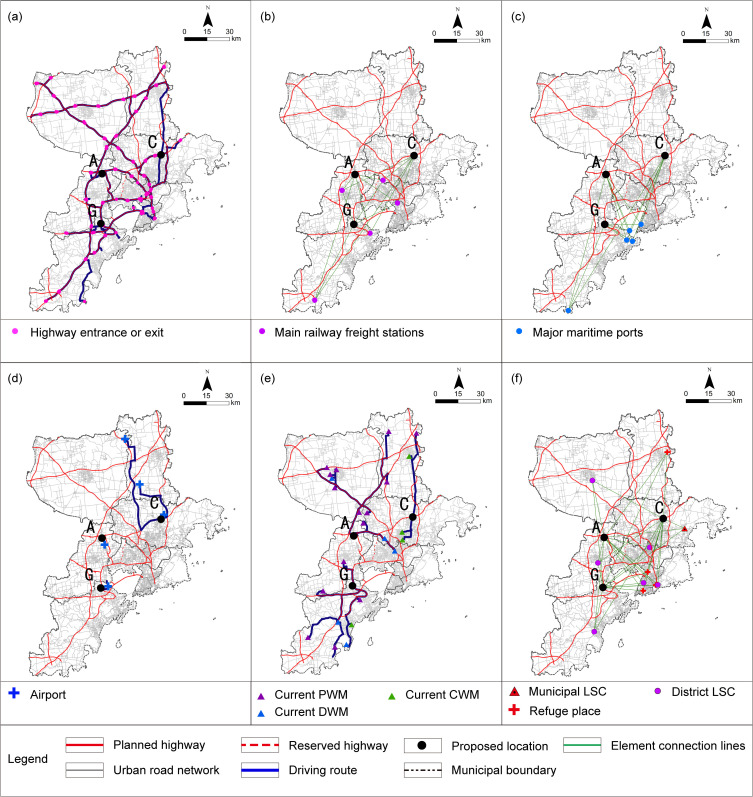
The analysis process of evaluation factors for T1, T3 to T5, F1-1 and F1-2 under the elastic principle. (a) T1: The driving time to the nearest expressway entrance and exit; (b) T3: ADT with railway freight stations; (c) T4: ADT with major port facilities; (d) T5: The driving time to the nearest airport; (e) F1-1: The nearest large-scale APWM driving time; (f) F1-2: ADT with the logistics support facilities. LSC, Logistics Support Center. Caption credit: This figure was created by the authors using ArcGIS 10.8.1, Adobe Illustrator 2023.

GIS technology was utilized to construct an OD cost matrix to calculate the average travel time for the elastic criteria associated with L1, P1, P2, T3, T4, T5, and F1-2. The requirements of Qingdao and cities along the Yellow River Basin agricultural products and agricultural materials circulation corridor to build agricultural products circulation platform [64], combined with Qingdao coastal location and road circulation path, the L1-1 criterion is derived by computing the average driving time from the location to the intersection points where the municipal boundary line meets each intercity expressway. In the calculation of the P1 criterion, given the average vehicle speed of 20.7 km/h [65] during peak hours on weekdays in Qingdao, it takes approximately 10 minutes to cover a radius of roughly 3 kilometers. Taking into account the principle of proximity-based collaboration, this estimate is adopted as the permissible coverage area for the relevant industrial cluster. For L2, T1, T5, and F1-1, the nearest facility point running time was determined. The commuting time for T2-2 is estimated by calculating the travel distances covered via subway and bus, combined with the average speeds associated with each mode of transportation. The subway network was constructed based on the government-approved report [66]，with an average operating speed of 44.83 km/h derived from the operator’s official statistical report [67]，Bus routes were verified using Baidu Maps Navigation, and the average bus speed was determined based on the peak-hour average speed of 16 km/h specified in the government-issued 2025 annual transportation plan [68]. The peak-hour speed is adopted to reflect the concentration of major trading activities in large wholesale markets during the morning rush hour, thereby fully accounting for the traffic congestion likely to occur during their peak operating periods. For T2-1, S1, S2, and F2, corresponding data were used for map-based measurements. The impedance values and their parameter sources used in the commuting evaluation criteria are summarized and explained in Table 6. All GIS-based parameter calculations were conducted within the CGCS2000 coordinate system Fig 10–12. The analysis of certain criteria is presented in Figs 10–12. Additional calculation principles are elaborated in Table 7.

**Table 6:** The impedance definition of the commuting type evaluation criteria.

Criteria code	OD impedance definition	Parameter source
**L1, P1, P2, T3, T4, F1-2**	The average time spent driving on expressway and urban roads(include urban expressway, trunk roads, secondary trunk roads).	Annual statistical data combined with design specification data.
**L2, T1, T5, F1-1**	The shortest time spent driving on expressway s and urban roads(include urban expressway, trunk roads, secondary trunk roads).	Annual statistical data combined with design specification data.
**T2-2**	Commuting time by subway and bus.	Annual average speed of metro and planned average speed of urban buses.

**Table 7:** Calculation of evaluation index based on the principle of elasticity.

Criteria	Object of analysis	Analysis basis	Proposed location selection			Units
			A1	C1	G1	
**L1-1**	The intersection point of Qingdao municipal boundary line and the planned Intercity expressway	TSP, CRTSP, CTSP	60.15	71.66	70.78	min
**L1-2**	The locations of towns and districts involved in the production areas of advantageous agricultural products, municipal-level exemplary family farms and professional cooperatives, and planned fisherman’s docks	SPFM, 14th ARMD, PLRF	56.23	63.90	67.48	
**L1-3**	Specialized origin market has been operated	SPFM, SPCN, PCMS and market research	52.16	66.58	69.54	
**L1-4**	Planned central farmers market	SPFM	44.45	51.13	40.92	
**L1-5**	Planned community farmer’s market		45.56	54.17	41.41	
**L1-6**	Major Supermarket POI	POI	43.63	51.00	47.32	
**L2**	Location of the municipal government	POI	51.70	52.36	40.58	min
**P1**	The area enclosed by the industrial control line of the territorial space planning covering more than 10 POI of food processing and cold chain logistics enterprises within a radius of 3 kilometers	Industrial control line in TSP, POI	35.26	44.87	44.40	min
**P2**	The “14th Five-Year Plan” for agricultural product processing industry clusters	14th DAPP1	38.94	49.41	57.80	min
**T1**	The nearest expressway entrance and exit	CTSP, POI	0.70	2.64	3.46	min
**T2-1**	Length along the location selection scope	TSP, CRTSP	858	1027	1568	m
**T2-2**	Qingdao Municipal Government, Qingdao Railway Station, Qingdao North Railway Station, Qingdao West Railway Station, Qingdao Cruise Home Port, Jiaodong International Airport	TURT, Baidu Maps Bus Routes	90.02	129.79	112.95	min
**T3**	Jiaozhou Container Center Station, Loushan Station, Huangdao Freight Station, Jimo Station, Dongjiakou South Station	Urban integrated transportation planning in TSP	40.37	60.27	37.24	min
**T4**	Aoshan Bay Port, Old City Port, Huangdao Port, Qianwan Port, Haixi Bay Port.		57.11	77.45	34.33	min
**T5**	Jiaodong International Airport, Laixi, Jimo, Pingdu, West Coast General Navigation Airport		9.41	10.56	10.61	min
**S1**	The scale of expandable commercial, warehousing and industrial land in the surrounding area	Land use planning in TSP and CRTSP	51.90	45.88	16.96	ha
**S2**	The scale of warehousing, logistics and food processing industrial land within 1 km of the surrounding area	The status of the class data in TSP	19.02	2.62	3.05	ha
**S3**	Regional benchmark land price	Qingdao 2019 benchmark land price inquiry system	240	330	300	Yuan/㎡
**S4**	Ground attachment	Satellite image survey	55728	183181	3599	㎡
**S5-1.**	Slope of the site	Construction land suitability evaluation results in TSP, open source DEM data	98.67	99.76	98.86	%
**S5-2.**	The scale of park green space is planned within 0.8 km	Land use planning in CRTSP	6.64	0.41	29.91	ha
**F1-1**	Current position of large-scale APWM	SPFM, SPCN, POI	17.47	18.61	20.17	min
**F1-2**	Planning location of city and district level logistical support center and emergency relief materials storage warehouse	Municipal disaster prevention and mitigation planning in TSP	46.26	49.63	48.25	min
**F2-1**	Planning the nearest residential area, large public service facilities	Infrastructure and land use planning in TSP and CRTSP	1497	1405	1103	m
**F2-2**	Hanjia sewage treatment plant, Automobile gas Gate station and LNG storage and distribution station, Wangtai Gas Gate station		2731	1743	1107	m

### Determination of comprehensive weight

#### Weight analysis based on AHP method.

Based on the pairwise comparison matrices of the criteria in Table 8, a panel of experts with diverse professional backgrounds—spanning government management, operational investment, cost control, industrial planning, territorial and spatial planning, architectural design, and potential merchants—was convened to assign weights to the 16 types of secondary criteria listed in Table 5, and allocate values to the sub-classification criteria under some secondary criteria based on the specific requirements of different market types, including production areas, consumption areas, and distribution areas, to construct a comprehensive matrix, refer to S1 Table, All individuals participating in the survey were informed of the research objectives and provided oral consent prior to participation. To protect privacy, we have anonymized all the experts. Consistency tests were performed separately using SPSSAU, with the consistency ratio (CR < 0.1). The effective matrix was then summarized using the geometric mean method, and the AHP weight values for the secondary classification and its sub-classification judgment matrix were calculated by inputting the data into the SPSSAU system (resulting CR = 0.031 < 0.1). Finally, pairwise multiplication was conducted to calculate the AHP weight values for all criteria $w_{a}$, as presented in Table 9.

**Table 8:** The importance score of criteria i compared to j.

Score index	Definition
**1**	Equal importance (EI)
**3**	Slightly more importance (SM)
**5**	High importance (HI)
**7**	Very high importance (VH)
**9**	Absolutely more importance (AM)
**2、4、6、8**	The median value of the above adjacency judgments
**1/a**_**ij**_	Inverse comparison between factor i and factor j

**Table 9:** Weight and comprehensive weight calculation of AHP-entropy weight method.

SC	AHP Weight Calculation								Entropy Weight		Comprehensive Weight(${𝐰}_{j}$)		
	Criteria Weight					${𝐰}_{a}$							
	Weighted Value	SSC	PWM	DWM	CWM	PWM	DWM	CWM	P/N Orientation	${𝐰}_{e}$	PWM	DWM	CWM
**L1**	0.149	L1-1	0.176	0.184	0.071	0.026	0.027	0.010	N	0.057	0.036	0.038	0.015
		L1-2	0.178	0.115	0.043	0.026	0.017	0.006	N	0.037	0.023	0.015	0.006
		L1-3	0.393	0.253	0.082	0.058	0.038	0.012	N	0.047	0.065	0.042	0.014
		L1-4	0.142	0.259	0.359	0.021	0.039	0.053	N	0.029	0.015	0.027	0.038
		L1-5	0.073	0.122	0.276	0.011	0.018	0.041	N	0.029	0.007	0.013	0.029
		L1-6	0.038	0.067	0.170	0.006	0.010	0.025	N	0.031	0.004	0.007	0.020
**L2**	0.095								N	0.061	0.129	0.138	0.142
**P1**	0.070								N	0.062	0.097	0.104	0.106
**P2**	0.049								N	0.033	0.036	0.038	0.039
**T1**	0.079								P	0.038	0.068	0.073	0.074
**T2**	0.035	T2-1	0.648			0.023			P	0.041	0.021	0.023	0.023
		T2-2	0.352			0.012			N	0.033	0.009	0.010	0.010
**T3**	0.036								N	0.028	0.022	0.024	0.025
**T4**	0.054								N	0.032	0.039	0.042	0.043
**T5**	0.072								N	0.062	0.101	0.108	0.111
**S1**	0.045								P	0.028	0.029	0.031	0.031
**S2**	0.017								P	0.066	0.026	0.027	0.028
**S3**	0.030								N	0.036	0.024	0.026	0.027
**S4**	0.020								N	0.029	0.013	0.013	0.014
**S5**	0.010	S5-1.	0.261			0.003			P	0.046	0.003	0.003	0.003
		S5-2.	0.739			0.007			P	0.043	0.007	0.008	0.008
**F1**	0.134	F1-1	0.692			0.093			P	0.033	0.069	0.074	0.076
		F1-2	0.308			0.041			N	0.034	0.031	0.033	0.034
**F2**	0.105	F2-1	0.263			0.028			P	0.028	0.018	0.019	0.019
		F2-2	0.737			0.078			P	0.034	0.060	0.064	0.066

SC, Secondary Criteria; SSC = Sub-classification Criteria; P, Positive; N, Negative.

#### Weight analysis based on entropy weight method.

A judgment matrix of m schemes and n evaluation indicators is constructed based on the data in Table 7, and positive and negative dimensional processing is carried out according to their relevant attributes, and the processed data is entered into the SPSSAU system for analysis. The steps are as follows:

First, the evaluation matrix X is constructed as below:


$$X={\left(x_{ij}\right)}_{n\times m}$$ (1)

where I=1, 2, …, n； j=1, 2, …, m.

Then the data was standardized and the forward indicators were calculated, as shown in Equation 2. The negative indicator is calculated, as shown in Equation 3. In order to avoid the situation that some data after standardization has a low value, H = 0.01 is taken for translation processing as shown in Equation 4.


$$r_{ij}=\frac{x_{ij}-min(x_{ij})}{max(x_{ij})-min(x_{ij})}$$ (2)

$$r_{ij}=\frac{max(x_{ij})-x_{ij}}{max(x_{ij})-min(x_{ij})}$$ (3)

$$\overset{\prime }{\underset{ij}{r}}=r_{ij}+H$$ (4)

Where $r_{ij}$ is the standardized processing of the positive and negative vectors to avoid the influence of different measurement units of the elements, and H is used as a translation vector to prevent the occurrence of zero values.

Finally, the SPSSAU system is used to calculate the entropy weight in Table 9.

#### Comprehensive weight calculation and analysis of calculation results.

The comprehensive weight of AHP+ entropy weight method in Table 9 is calculated using Equation 5. Meanwhile, the standardized values calculated by Equation 2,3 are used to obtain the comprehensive scores of different evaluation criteria by using linear weighting method calculated as Equation 6, which is obtained in Table 10.

**Table 10:** Calculation of evaluation results.

Proposed location selection	PWM	DWM	CWM
**A1**	0.729	0.721	0.705
**C1**	0.165	0.160	0.156
**G1**	0.379	0.401	0.442


$$w_{j}=\frac{w_{a}w_{e}}{\sum w_{a}w_{e}}$$ (5)

Where $w_{a}$ denote the AHP weight for criterion j and $w_{e}$ denote the entropy weight; We calculate the comprehensive weight $w_{j}$ by summing the products of the two types of weights and normalizing them.


$$z_{i}=\sum_{j=\text{1}}^{n}w_{j}\overset{\prime }{\underset{ij}{r}}$$ (6)

According to the calculation, A1 location selection scheme is obviously superior to other schemes, especially in the layout of the production market, followed by the distribution market and sales market. G1 was the second choice after A1, while C1 had the lowest score. To analyze the results more clearly, this time the partial comprehensive method was used to analyze the contribution rate of the scores under the market weights of each type, in order to determine the contribution value of each element to the final score. Equation 7 represents the contribution value of the i criterion, and Equation 8 is the contribution degree ratio $R_{i}$ of the i criterion.


$$C_{i}=\Delta_{i}*w_{j}$$ (7)

Where, $\Delta_{i}$ denotes the difference in standardized scores $\overset{\prime }{\underset{ij}{r}}$ under the i-th factor evaluation between two locations, and $w_{j}$ represents the corresponding comprehensive weight.


$$R_{i}=\left(C_{i}/\Delta_{t}\right)*\text{100}\%$$ (8)

Where, $\Delta_{t}$ denotes the sum of the differences in standardized scores $\overset{\prime }{\underset{ij}{r}}$ across all factor evaluations between two locations. A positive $R_{i}$ value indicates the degree to which the criterion contributes to the location’s score, whereas a negative value reflects the extent to which it negatively impacts the score.

**Fig 13:**
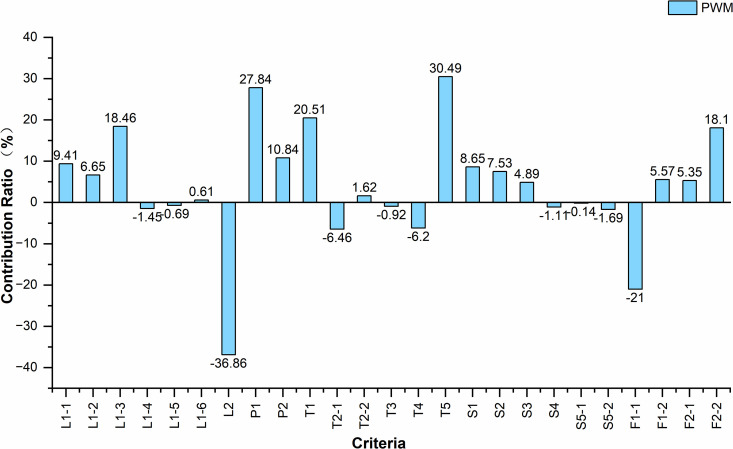
Contribution rate of criteria under the weight of PWM.Caption credit: This figure was created by the authors using OriginPro 2024b.

**Fig 14:**
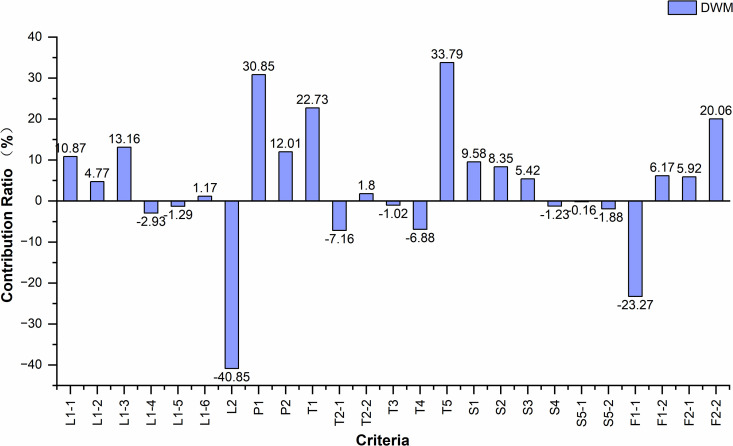
Contribution rate of criteria under the weight of DWM. Caption credit: This figure was created by the authors using OriginPro 2024b.

**Fig 15:**
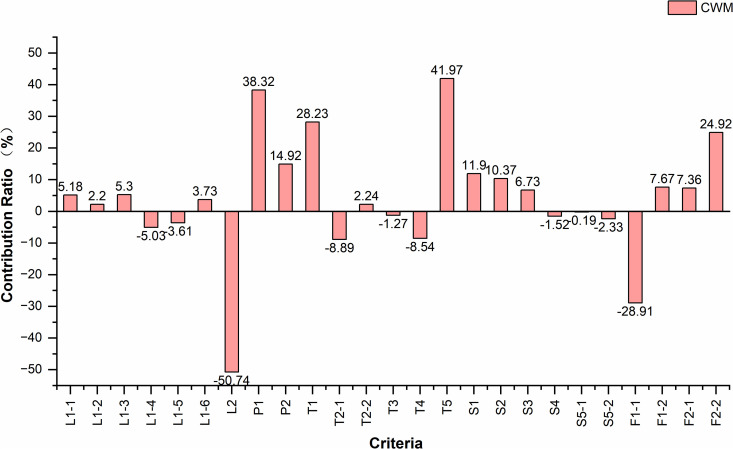
Contribution rate of criteria under the weight of origin CWM. Caption credit: This figure was created by the authors using OriginPro 2024b.

Given that A1 and G1 represent the optimal and suboptimal location alternatives, respectively, a comparative analysis was conducted to examine the influencing factors between these two schemes. The results under the comprehensive weight of the origin market are presented in Table 11, while the corresponding analytical outcomes under all attribute weights are illustrated in Figs 13–15.

**Table 11:** Evaluation results of contribution rate.

Criteria	${𝐰}_{j}$(PWM)	Score of A1	Score of G1	$\Delta_{i}$(PWM)	${𝐂}_{i}$(PWM)	${𝐑}_{i}$(PWM)
**L1-1**	0.036	1.010	0.086	0.924	0.033	9.4%
**L1-2**	0.023	1.010	0.010	1.000	0.023	6.6%
**L1-3**	0.065	1.010	0.010	1.000	0.065	18.5%
**L1-4**	0.015	0.664	1.010	−0.346	−0.005	−1.4%
**L1-5**	0.007	0.685	1.010	−0.325	−0.002	−0.7%
**L1-6**	0.004	1.010	0.509	0.501	0.002	0.6%
**L2**	0.137	0.066	1.010	−0.944	−0.129	−36.9%
**P1**	0.103	1.010	0.059	0.951	0.098	27.8%
**P2**	0.038	1.010	0.010	1.000	0.038	10.8%
**T1**	0.072	1.010	0.010	1.000	0.072	20.5%
**T2-1**	0.023	0.010	1.010	−1.000	−0.023	−6.5%
**T2-2**	0.010	1.010	0.433	0.577	0.006	1.6%
**T3**	0.024	0.874	1.010	−0.136	−0.003	−0.9%
**T4**	0.041	0.482	1.010	−0.528	−0.022	−6.2%
**T5**	0.107	1.010	0.010	1.000	0.107	30.5%
**S1**	0.030	1.010	0.010	1.000	0.030	8.6%
**S2**	0.027	1.010	0.036	0.974	0.026	7.5%
**S3**	0.026	1.010	0.343	0.667	0.017	4.9%
**S4**	0.013	0.720	1.010	−0.290	−0.004	−1.1%
**S5-1.**	0.003	0.010	0.184	−0.174	0.000	−0.1%
**S5-2.**	0.008	0.221	1.010	−0.789	−0.006	−1.7%
**F1-1**	0.074	0.010	1.010	−1.000	−0.074	−21.0%
**F1-2**	0.033	1.010	0.420	0.590	0.020	5.6%
**F2-1**	0.019	1.010	0.010	1.000	0.019	5.3%
**F2-2**	0.063	1.010	0.010	1.000	0.063	18.1%
**Comprehensive evaluation**	1.000	0.739	0.389	0.351	0.351	100.0%

Based on the analysis results, under the three weighting scenarios corresponding to origin markets, distribution centers, and destination markets, the three criteria contributing most to A1’s higher score relative to G1 are T5, P1, and T1, while those exerting the greatest negative impact are L2, F1-1, and T2-1. This indicates that A1 holds significant advantages in proximity to airport facilities, planned concentrated industrial development zones, and expressway exits, whereas G1 demonstrates comparative strengths in closeness to the city center, maintain an appropriate distance from existing markets., and access to major urban roads.

#### Analysis of the relevance of criteria and concentration of weights.

As the location selection process has resulted in three candidate areas following a stepwise screening procedure, and considering the limitation in sample size (n = 3), the spatial correlation among location selection criteria based on commuting time and the OD cost matrix is assessed using the Spearman rank correlation coefficient, as defined in Equation 9. The potential risk is quantified by the degree of weight concentration. Based on the analysis of the original data in Table 7, the performance of each criterion is ranked according to relative superiority in Table 12, where higher values of positive-direction indicators indicate better performance, while lower values of negative-direction indicators are preferable. Rank 1 denotes the best performance, rank 2 the second best, and rank 3 the worst.

**Table 12:** Ranking of location selection sample criteria scores.

Criteria	Ranking		
	A1	C1	G1
**L1**	1	3	2
**L2**	1	2	3
**P1**	1	2	3
**P2**	1	2	3
**T1**	1	2	3
**T2-2**	1	3	2
**T3**	1	3	2
**T4**	2	3	1
**T5**	1	2	3
**F1-1**	3	2	1
**F1-2**	1	3	2

The calculation of L1 takes the average of the values of L1-1 and L1-2.


$$p=\text{1}-\frac{\text{6}\sum \overset{\text{2}}{\underset{i}{d}}}{n(n^{\text{2}}-\text{1})}$$ (9)

Where, the Spearman rank correlation coefficient p primarily reflects the rank-based association within the sample, rather than the statistical significance typical of large samples; n = 3 denotes the sample size, and $d_{i}$ represents the difference in ranks.

**Fig 16:**
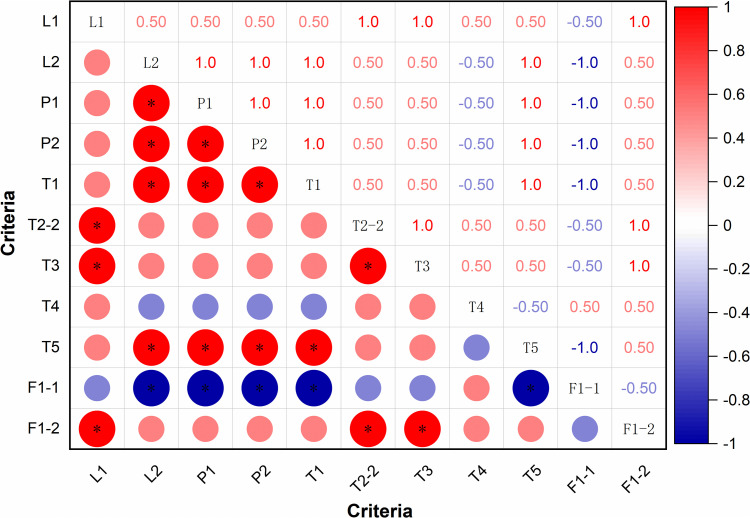
Heatmap of the relevance of some evaluation criteria. * indicates that the P value ≤ 0.05. Caption credit: This figure was created by the authors using OriginPro 2024b.

The feature matrix derived from the results was processed using the Spearman rank correlation to compute the correlation coefficients among the various evaluation criteria, and a corresponding heatmap is presented in Fig 16.

Through analysis, it is evident that L1 exhibits a significant positive correlation with T2-2, T3, and F1-2; L2 with P1, P2, T1, and T5; P1 with P2, T1, and T5; P2 with T1 and T5; T1 with T5; T2-2 with T3 and F1-2; and T3 with F1-2. Additionally, F1-1 shows a significant negative correlation with L2, P1, P2, T1, and T5. The average criteria exhibiting higher correlations demonstrate varying degrees of weight concentration, as detailed in Table 13. These patterns are closely associated with the traffic-related geographical characteristics considered during the location selection process. The corresponding heatmap is presented in (Fig 16).

**Table 13:** The concentration ratio of the weights of correlative criteria.

Correlative Criteria	Comprehensive weight proportion		
	PWM	DWM	CWM
**L1, T2-2, T3, F1-2**	27.06%	26.37%	24.61%
**L2, P1, P2, T1, T5**	45.64%	46.08%	47.18%
**F1-1, L2, P1, P2, T1, T5**	53.01%	53.51%	54.79%

### Sensitivity analysis of partial elasticity criteria

During the process of determining objective weights, the average speed for each road network class used in the OD cost matrix is established based on multi-year statistical data and in conjunction with road design standards, ensuring relative stability. However, the metro speed, bus speed, and peak-hour motor vehicle operating speed employed in the T2-2 and P1 evaluation criteria are derived from specific-year statistics or planning projections, exhibiting a certain degree of variability. These factors directly influence the OD impedance associated with the T2-2 criterion and the OD path under the P1 criterion. Therefore, a sensitivity analysis is conducted on these three types of influencing factors. By assuming adjustments of ±10% for metro speed, ± 20% for bus speed, and an increase or decrease of 5 minutes in commuting time within the 10-minute industrial cluster radius, a perturbation analysis is performed on the relevant parameters.

**Fig 17:**
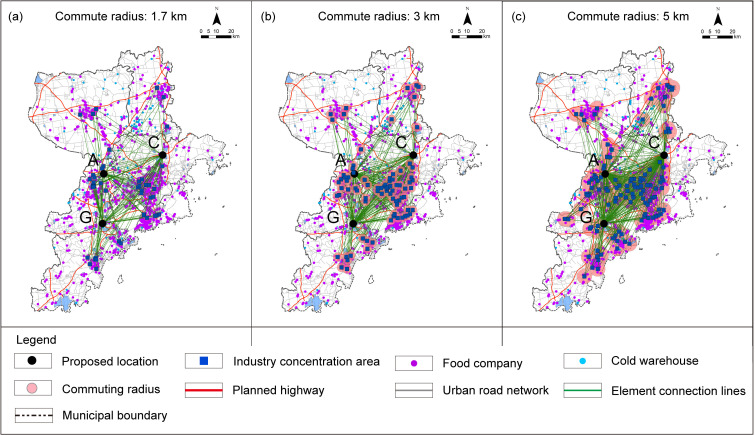
P1 criterion perturbation analysis Caption credit: This figure was created by the authors using ArcGIS 10.8.1, Adobe Illustrator 2023.

During the perturbation analysis of the P1 value, with commuting radius durations set at 5, 10, and 15 minutes, the corresponding commuting radii were converted to 1.7 km, 3.0 km, and 5.0 km, respectively. The GIS point density analysis function was applied to identify functional aggregation areas within these radii where the number of POIs for food companies and cold storage facilities exceeded 10. These identified areas were subsequently overlaid with the industrial development zones defined by national land space planning. The centroid of each overlapping area was designated as the destination point of the respective industrial aggregation zone. Under the three commuting time thresholds, the numbers of destination points meeting the criteria were 46, 84, and 114, respectively. The average commuting distance between the proposed market location and these destination points was calculated using the OD cost matrix (Fig 17), from which the statistical values for the P1 location selection criterion were derived.

The calculation and statistical results of the relevant criteria after the disturbance of the above elements are shown in Table 14.

**Table 14:** Calculation of evaluation results.

Criteria	Parameters and disturbance values			Statistical values of candidate location factors (min)		
	Definition of impedance	parameter values	Direction and amplitude of disturbance	A1	C1	G1
**P1**	Commute time within the radius of industrial clusters	5 min	−5 min	34.82	49.00	43.76
		10 min	Original value	35.26	44.87	44.40
		15 min	+5 min	36.61	48.45	43.53
**T2-2**	Average speed of the subway	40.35 km/h	−10%	98.6	139.41	121.53
		44.83 km/h	Original value	90.02	129.79	112.95
		49.31 km/h	+10%	83	121.91	105.92
	Average speed of buses during peak hours	12.8 km/h	−20%	93.21	140.57	121.85
		16 km/h	Original value	90.02	129.79	112.95
		19.2 km/h	+20%	87.89	122.60	107.01

Following the perturbation, the calculated comprehensive weight values for P1 and T2-2 are presented in Table 15. It can be observed that under a ± 5-minute variation in the industrial cluster commuting radius, the comprehensive weight changes within the range of −40% to −43%. This change is primarily attributed to the altered average commuting time resulting from shifts in destination points within the industrial concentration area, which reduces the comprehensive weight of this criterion. In contrast, perturbations in bus and subway average speeds lead to proportional changes in commuting time for T2-2, without affecting the corresponding weight coefficient.

**Table 15:** Calculation of the comprehensive weight value after disturbance.

Criteria	Parameters and disturbance values			Comprehensive weight		
**Definition of impedance**	**parameter values**	**Direction and amplitude of disturbance**	**PWM**	**DWM**	**CWM**
**P1**	Commute time within the radius of industrial clusters	5 min	−5 min	0.061	0.062	0.063
		10 min	Original value	0.103	0.104	0.106
		15 min	+5 min	0.059	0.059	0.061
**T2-2**	Average speed of the subway	40.35 km/h	−10%	0.010	0.010	0.010
		44.83 km/h	Original value	0.010	0.010	0.010
		49.31 km/h	+10%	0.010	0.010	0.010
	Average speed of buses during peak hours	12.8 km/h	−20%	0.010	0.010	0.010
		16 km/h	Original value	0.010	0.010	0.010
		19.2 km/h	+20%	0.010	0.010	0.010

When the weight of P1 varies, the comprehensive scores of the three location selection alternatives exhibit only minor fluctuations, with no impact on the overall ranking. The comprehensive scores and corresponding change rates for each site under different P1 market weight scenarios are presented in Table 16.

**Table 16:** Calculation of the comprehensive weight value after disturbance.

Proposed location selection	Change criteria	Scene after change	Comprehensive scores			The rate of change in the comprehensive score		
			PWM	DWM	CWM	PWM	DWM	CWM
**A1**	P1	Commute time radius: 5 minutes	0.717	0.708	0.691	−1.72%	−1.82%	−2.01%
**C1**			0.173	0.168	0.164	4.65%	4.69%	4.81%
**G1**			0.414	0.438	0.481	9.22%	9.05%	8.86%
**A1**	P1	Commute time radius: 15 minutes	0.716	0.707	0.690	−1.83%	−1.92%	−2.13%
**C1**			0.174	0.168	0.164	4.92%	4.97%	5.09%
**G1**			0.416	0.441	0.484	9.96%	9.77%	9.56%

## Comprehensive weight optimization based on GA

After the conventional application of the aforementioned methods to calculate comprehensive weights, it is recognized that not all indicators have been subjected to perturbation, and the correlation and weight concentration among certain indicators are taken into account. Furthermore, in multi-criteria evaluation, subjective weighting methods (e.g., AHP) rely on expert judgment but are susceptible to subjective bias, whereas objective weighting methods (e.g., the entropy weight method) depend on data characteristics yet exhibit limited reliability in small-sample settings. GA has been employed in several studies for location selection and optimization of transportation and logistics facilities, yielding favorable outcomes [69–72].For the high-dimensional, small-sample scenario of “25 evaluation indicators and 3 samples” addressed in this study, a GA-optimized AHP–CV (coefficient of variation) integrated weighting approach is proposed to enhance the validation and optimization of comprehensive weights for the three market types. In this method, AHP-derived weights reflect subjective expertise, CV-derived weights capture the objective variability within limited samples, and the two are adaptively combined through GA global optimization, thereby balancing theoretical rationality with data-driven adaptability in the evaluation process.

### Subjective weights and objective weights

The AHP weights were determined through the pairwise comparison matrix derived from expert assessments of indicator importance, as presented in Table 9. This study adopts the AHP weight vector that has passed the consistency test (CR < 0.1).


$$w_{a}=[w_{a\text{1}},w_{a\text{2}},\ldots ,w_{a\text{25}}]$$

Satisfy $\;\sum_{i=\text{1}}^{\text{25}}w_{ai}=\text{1}$ and $w_{ai}\geq \text{0}$

Where $w_{ai}$ represents the AHP weight of the i-th indicator.

For the small-sample scenario involving three samples, the CV is adopted as the dispersion measure to address potential objective weight distortion, replacing the entropy weight method. The calculation formula is presented in Equation 10.


$${CV}_{i}=\frac{S_{i}}{{\overset{―}{x}}_{i}}(\text{i}=\text{1},\;\text{2},...,\text{ 25})$$ (10)

Where, ${\overset{―}{x}}_{i}=\frac{\text{1}}{\text{3}}\sum_{k=\text{1}}^{\text{3}}x_{ki}$ represents the sample mean of the i-th indicator, and $S_{i}=\sqrt{\frac{\text{1}}{\text{2}}\sum_{k=\text{1}}^{\text{3}}{\left(x_{ki}-{\overset{―}{x}}_{i}\right)}^{\text{2}}}$ is the sample standard deviation (with a degree of freedom of 1, suitable for small samples).

The objective weights are derived by normalizing the CV using Equation 11.


$$w_{ci}=\frac{CV}{\sum_{j=\text{1}}^{\text{25}}{CV}_{j}}$$ (11)

After correction, it should satisfy: 0.01≤$w_{ci}$ ≤0.15 and $\sum_{i=\text{1}}^{\text{2}\text{5}}w_{ci}=\text{1}$. The correction procedure is as follows: weights below 0.01 are set to 0.01, those above 0.15 are set to 0.15, and the resulting vector is subsequently re-normalized to mitigate the influence of extreme values in small samples.

### GA-optimized comprehensive weight

The comprehensive weight $w=\left[w_{\text{1}},w_{\text{2}},\ldots w_{\text{25}}\right]$, needs to be close to both the subjective weight of AHP and the objective weight of CV simultaneously. The objective function is defined as Equation 12:


$$\text{min}\;\text{F}(\text{w})=\lambda_{\text{1}}\times \text{D}\left(\text{w},{\text{w}}_{\text{a}}\right)+\lambda_{\text{2}}\times \text{D}\left(\text{w},{\text{w}}_{\text{c}}\right)$$ (12)

Where: $D\left(w,w_{a}\right)=\sum_{i=\text{1}}^{\text{25}}\left|w_{i}-w_{ai}\right|$ is the Manhattan distance between the comprehensive weight and the AHP weight (subjective deviation). $\text{D}\left(\text{w},{\text{w}}_{\text{a}}\right)=\sum_{\text{i}=\text{1}}^{\text{25}}\left|{\text{w}}_{\text{i}}-{\text{w}}_{\text{ci}}\right|$ is the Manhattan distance between the comprehensive weight and the CV weight (subjective deviation).

$\lambda_{\text{1}}=\text{0}.\text{6}$, $\lambda_{\text{2}}=\text{0}.\text{4}$ is the weighting coefficient (since the objective weighting only took values from three location samples, subjective weighting is emphasized here).

The constraint conditions:


$$\left\{ \begin{array}{c} \sum_{\text{i}=\text{1}}^{\text{25}}{\text{w}}_{\text{i}}=\text{1}\;(\text{Unity},\text{ allowing for a tolerance of }\pm \text{0}.\text{01})\\ \text{0}.\text{0}\text{1}\leq w_{i}\leq \text{0}.\text{1}\text{5}\;(\text{Avoid excessive concentration or dispersion of weights})\\ w_{i}\geq \text{0}\; \end{array}\right.\;$$

In the GA optimization process, real-number coding is employed. The chromosome represents a 25-dimensional weight vector w. The initial population consists of 150 randomly generated weight vectors ($w_{i}\in [\text{0}.\text{0}\text{1},\text{0}.\text{1}\text{5}]$) that satisfy the prescribed constraints and are subsequently normalized.

The fitness function, as defined in Equation 13, is constructed such that a lower objective function value corresponds to higher fitness.


f(w)=1/(1+F(W)) (13)

Genetic operations:

Selection: Tournament selection (randomly select 3 individuals and retain the one with the highest fitness);

Crossover: Simulated binary crossover (SBX), crossover probability 0.9, distribution index η = 20;

Mutation: Polynomial mutation, mutation probability 0.03, distribution index η = 20;

Termination condition: Iterate until the objective function converges or remains stable without significant change for 30 consecutive generations (change < 1 × 10^−6^).

Optimization result: The comprehensive weight $w^{*}$ obtained after GA optimization should satisfy:$w^{*}=arg\;minF(w)$

Through iterative optimization, the final comprehensive weight retains the subjective rationality of AHP, absorbs the objective data characteristics of CV, and has a balanced weight distribution (standard deviation < 0.05).

The consistency test was performed using the Spearman rank correlation coefficient, as defined in Equation 9, to calculate the correlation between the comprehensive weight and the AHP weight.


$$p=\text{1}-\frac{\text{6}\sum \overset{\text{2}}{\underset{i}{d}}}{n(n^{\text{2}}-\text{1})}$$ (14)

Where, $d_{i}$ denotes the rank difference of the i-th indicator under the two weighting schemes, where n = 25. A Spearman correlation coefficient p > 0.6 indicates acceptable consistency between the two sets of weights. For the stability test, the comprehensive weight is slightly perturbed by ±5%, and the resulting change rate of the evaluation outcome is calculated using Equation 15.


$$E_{rate}=\frac{\left|E_{d}-E_{o}\right|}{E_{o}}$$ (15)

If $E_{rate}$<10%, it indicates that the stability of the weight is acceptable.

**Fig 18:**
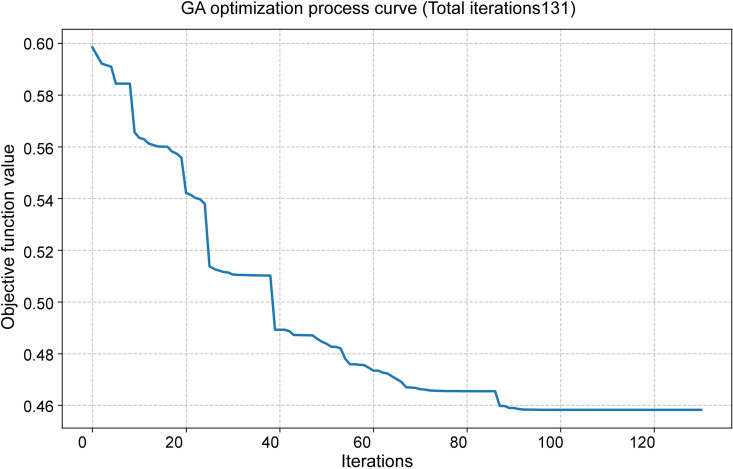
The curve of the GA optimization process for PWM weights. Caption credit: This figure was created by the authors using PyCharm 2025.1.1.1.

**Fig 19:**
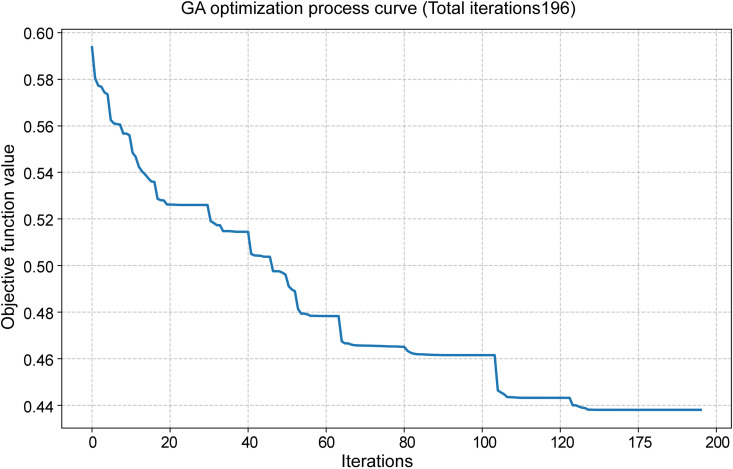
The curve of the GA optimization process for DWM weights. Caption credit: This figure was created by the authors using PyCharm 2025.1.1.1.

**Fig 20:**
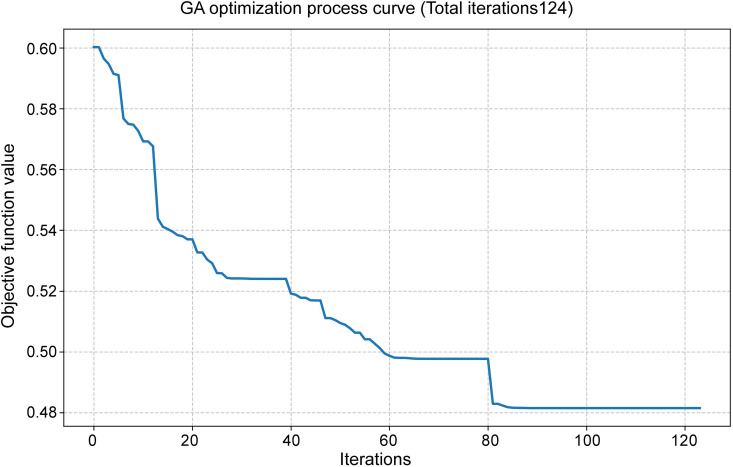
The curve of the GA optimization process for CWM weights. Caption credit: This figure was created by the authors using PyCharm 2025.1.1.1.

Following the aforementioned steps, Python was utilized to implement matrix operations via NumPy and statistical tests via SciPy, enabling efficient execution of GA operations such as crossover and mutation, as well as statistical analyses including Spearman’s rank correlation coefficient. Calculation curves were generated using Matplotlib Fig 18–20. The weights for production areas, sales areas, and distribution centers were optimized through GA, yielding updated objective and comprehensive weights in Table 17. Iterative computations across all market types satisfied the constraint conditions and demonstrated satisfactory convergence, as show in Table 18.

**Table 17:** The results of the objective weights $w_{c}$ and the comprehensive weight $w^{*}$ of various markets after GA optimization.

PWM		DWM		CWM	
${𝐰}_{c}$	${𝐰}^{*}$	${𝐰}_{c}$	${𝐰}^{*}$	${𝐰}_{c}$	${𝐰}^{*}$
0.012	0.036	0.012	0.027	0.012	0.019
0.012	0.028	0.012	0.010	0.012	0.012
0.019	0.042	0.019	0.040	0.019	0.014
0.014	0.025	0.014	0.032	0.014	0.054
0.017	0.014	0.017	0.023	0.017	0.026
0.010	0.011	0.010	0.016	0.010	0.012
0.017	0.072	0.017	0.068	0.017	0.076
0.016	0.064	0.016	0.067	0.016	0.060
0.024	0.030	0.024	0.036	0.024	0.032
0.078	0.066	0.078	0.079	0.078	0.086
0.040	0.022	0.040	0.045	0.040	0.023
0.027	0.021	0.027	0.022	0.027	0.022
0.034	0.043	0.034	0.036	0.034	0.036
0.048	0.045	0.048	0.053	0.048	0.047
0.010	0.064	0.010	0.028	0.010	0.059
0.061	0.046	0.061	0.064	0.061	0.062
0.143	0.059	0.143	0.023	0.143	0.063
0.020	0.020	0.020	0.027	0.020	0.030
0.144	0.031	0.144	0.025	0.144	0.029
0.010	0.015	0.010	0.022	0.010	0.012
0.149	0.045	0.149	0.029	0.149	0.020
0.010	0.079	0.010	0.087	0.010	0.080
0.010	0.035	0.010	0.047	0.010	0.021
0.019	0.019	0.019	0.028	0.019	0.032
0.055	0.069	0.055	0.067	0.055	0.074

**Table 18:** The result of the GA optimization process.

Characteristic value	PWM	DWN	CWM
**Total number of iterations**	131	196	124
**Coefficient of rank correlation**	0.7569	0.8669	0.7808
**Weight standard deviation**	0.0199	0.0204	0.0209
**Average rate of change in evaluation results**	1.76%	1.54%	2.22%
**The maximum rate of change**	4.48%	4.29%	4.04%

### Calculation of location selection results

Using the comprehensive weights optimized by the GA, the comprehensive scores for market location selection were recalculated according to Formula (16). The results, presented in Table 19, are consistent with the location selection recommendations obtained from the traditional calculation method shown in Table 10. Site A1 ranks first, followed by G1 and C1. This further confirms the accuracy and reliability of the location selection process under the established evaluation criteria.

**Table 19:** The location evaluation results after optimizing the comprehensive weight by GA.

Proposed Location Selection	PWM	DWM	CWM
**A1**	0.729	0.701	0.732
**C1**	0.190	0.226	0.209
**G1**	0.426	0.459	0.440


$$z_{i}=\sum_{j=\text{1}}^{n}w^{*}\overset{\prime }{\underset{ij}{r}}$$ (16)

## Discussion

### Discussion on criteria and weights.

**Fig 21:**
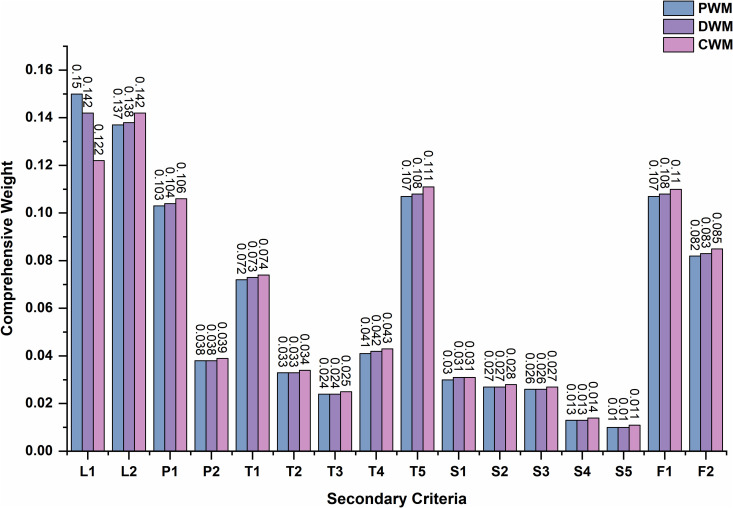
Comprehensive weight structure analysis. Caption credit: This figure was created by the authors using OriginPro 2024b.

Based on the analysis of comprehensive weights presented in Table 9 as show in (Fig 21), among the 16 secondary criteria, the top three for origin markets and distribution markets are L1, L2, and T5 (with F1 basic and T5 at an equivalent level), accounting for 39.40% and 38.82%, respectively. For destination markets, the leading criteria are L2, L1, and T5 (again with F1 basic and T5 at an equivalent level), collectively contributing 37.37%. This variation is primarily attributed to destination markets’ greater reliance on customer bases and population resources located in central urban areas. The subsequent three criteria—F1, P1, and F2—account for 29.17%, 29.45%, and 30.15%, respectively. It is evident that all market types exhibit substantial weighting toward factors such as connectivity between production and sales regions, proximity to the central urban area, and distance from airports. Additionally, sensitivity is observed regarding distance from similar markets, planned industrial concentration zones, and hazardous or polluted sources. The combined weight of the top six criteria exceeds 65% across all market categories.

In previous research, Uyanık et al. reviewed 35 studies on logistics facility location [29], and the most frequently mentioned location criteria were Cost (land, labor, investment, facility, operation, transportation, information), followed by Natural Resources (water, power and electric supply, weather, etc.), and then Proximity to railroad, highways, airports, and labor force. This study also provides a review of the selection and classification of location criteria in Table 1. In comparison with earlier findings, under the emerging context of new circulation patterns, cost considerations between production and sales locations remain the most influential factor. However, advances in engineering, environmental adaptation technologies, transportation infrastructure, and related fields have gradually reduced the significance of natural resource factors. Proximity to transportation networks and labor resources continues to play a critical role. Notably, with improvements in circulation efficiency and quality, the expanding reach of distribution networks, and the development of the low-altitude economy, the importance of proximity to airports has substantially increased.

Following a comprehensive evaluation of the candidate schemes, this study conducted perturbation analysis on selected evaluation criteria by varying average vehicle speed and commuting radius duration. Simultaneously, the comprehensive weights were optimized using a GA. Notably, none of these adjustments altered the initial ranking of location selection advantages and disadvantages. This outcome indicates, on one hand, that the selection criteria and their corresponding weight assignments in this study are relatively stable and representative. On the other hand, it reflects the intercorrelation among certain criteria and the concentration of their weights. Furthermore, these findings indirectly suggest that, within a specific regional context, there exist relatively favorable zones for locating large-scale agricultural product wholesale market logistics facilities—highlighting both theoretical significance and practical research value.

### Practical verification of location selection results

**Fig 22:**
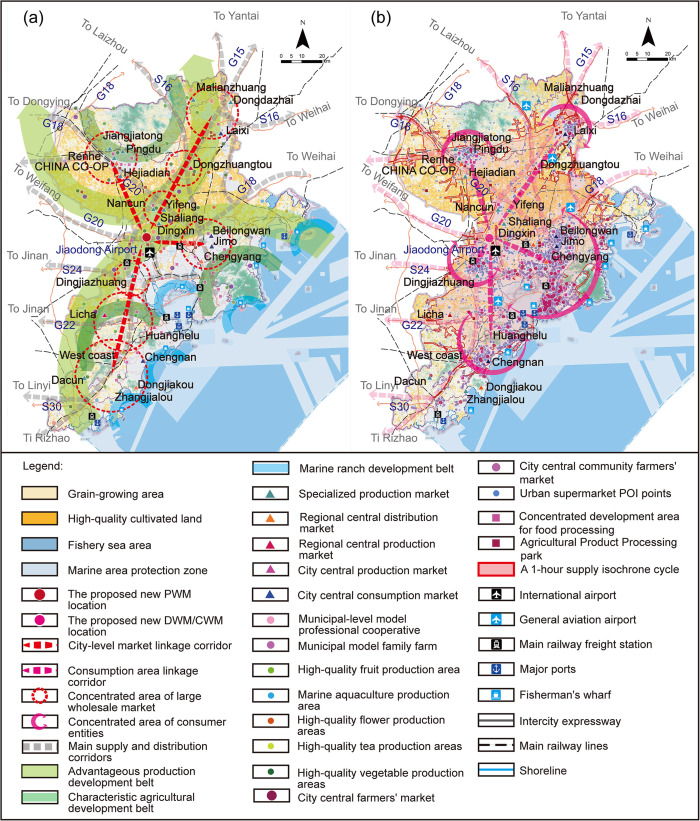
The municipal agricultural product circulation system under the A1 location selection scheme. (a) The PWM circulation system under the newly selected location A1; (b) The DWM and CWM circulation system under the newly selected location A1. Caption credit: This figure was created by the authors using Adobe Illustrator. The base map is sourced from the “Territorial Spatial Planning of Qingdao City (2021-2035)” (Public Display Version). Available from: http://www.jimo.gov.cn/zwgk/bmxxgk/zrzyj/zdgk/ghjh/kjgh/202110/t20211016_3637933.shtml.

Research on logistics centers and logistics markets facilitates scientific decision-making in facility location. Studies on location selection models, influencing criteria, and methodologies have yielded diverse outcomes. However, given the evolving trends of modern circulation, there remains a lack of sufficient research on aligning the location selection of large-scale APWM with the demands of modern circulation. This paper examines the novel characteristics associated with the transition from traditional to modern circulation, develops a quantitative location selection evaluation system tailored for modern circulation, thereby contributing new strategies and approaches for constructing an efficient modern circulation system for agricultural products. Through case studies, the paper draws conclusions on optimal location selection. Further analysis of the location selection conclusions for the proposed PWM, DWM, and CWM in the case city, in conjunction with the layout of territorial and spatial planning and industrial planning, indicates that:

As a state-level and backbone PWM for agricultural products, the A1 location is strategically situated at the intersection of the continuous development zones of the two dominant producing areas in Qingdao City. It maintains strong connectivity with the characteristic agricultural product development zone, the coastal marine pasture development zone, and the fisherman’s wharf. Emphasis is placed on establishing close linkages with specialized producing area wholesale markets. Positioned at the core distribution hub of eight key areas, it encompasses the existing large-scale agricultural product wholesale market, the “14th Five-Year Plan” agricultural product processing park, the food processing enterprise cluster zone, the municipal demonstration professional cooperative society, and the family farm cluster zone. This strategic positioning facilitates the formation of four linkage corridors that connect various market entities within the city. As can be seen from (Fig 22a).

As the location for a DWM or CWM: within the consumption network, it is positioned closest to the primary urban area. It is centrally located relative to the city’s farmers’ markets, community markets, and the main supermarket POI clustering zones. The one-hour supply isochrone established by this location selection essentially covers 81.36% of the total urban development boundary area. Proximity to key agricultural product production areas in the northwest, as well as aggregation zones such as Jiaozhou, Pingdu, and Laixi, facilitates efficient collection and supply of agricultural products throughout the city. Optimal public transport accessibility supports the establishment of an integrated wholesale market that combines travel, shopping, research, learning, and entertainment functions. As can be seen from (Fig 22b).

In terms of logistics transportation, the location is strategically located near the Jiaodong International Airport and the Jiaozhou “Belt and Road Initiative” multimodal international freight railway land-port. Situated in the relatively central area of the intercity expressway entrance and exit network’s radiation zone, it can establish a logistics circulation corridor system that connects domestic and international destinations, extends throughout the country, reaches the Shandong Peninsula, and covers the entire city.

### Expert discussion on the location selection results

To validate the reliability of the location selection outcomes derived from this study, the final location selection recommendations were submitted for review to a temporarily established expert review committee. This committee comprised specialists from the fields of market development, urban planning, logistics management, integrated transportation systems, and marketing, as well as representatives from key commercial stakeholders. The principal feedback and evaluations are summarized in Table 20.

**Table 20:** Opinions of experts of different types.

Expert type	Expert opinion
**Construction investment**	The location selection criteria comprehensively address various factors related to market investment and construction, demonstrating rigor and innovation. By providing differentiated location selection recommendations according to market type, they offer robust support for the early-stage planning and development of markets.
**Urban planning**	Through the innovative integration of multi-level market influencing factors with territorial spatial planning outcomes, the location selection approach not only fulfills operational requirements but also aligns with higher-level urban planning controls.
**Logistics management**	The incorporation of new circulation concepts enables the selected market locations to connect effectively with broader logistics networks, thereby enhancing circulation efficiency. During actual implementation, sufficient flexibility should be maintained to accommodate future developments in logistics systems.
**Integrated transportation**	Location selections for both market types fully account for access to large-scale transportation infrastructure. The established evaluation criteria are highly significant, enabling subsequent investment in transportation resource upgrades during later stages of market development based on predefined element standards.
**Merchant representative**	The location selection process thoroughly considers all stakeholders involved in production, sales, and supply chains, aiming to achieve balance across the entire commodity circulation system. In particular, by applying differential weighting, it captures the distinct preferences of different market types, ensuring that both production-oriented and sales-oriented markets are positioned in proximity to their most advantageous marketing resources.

### Social impact discussion

Accessibility and Equity: Under the new circulation system, large-scale agricultural product wholesale markets are closely linked to major suppliers and centralized purchasers, while also connecting diverse individuals through emerging transaction platforms such as internet-based systems and mobile applications. First, the protection of disadvantaged groups is comprehensively addressed in this study through indicators including public transportation accessibility, a 15-minute convenient delivery network, and spatial layouts compatible with e-commerce operations. In the event of major natural disasters or public health emergencies, the scope of vulnerable populations may expand to encompass the majority of urban residents. Consequently, the accessibility of emergency support measures plays a significant role in the location selection rationale of this research. Simultaneously, a 15-minute driving service radius is established as a key inflexible criterion for ensuring equitable distribution of market resources within urban areas.

Urban-Suburban and Urban-Rural Integration: The new circulation framework connects a broader spectrum of logistical resources. With continuous advancements in logistics information technology, infrastructure, and transportation systems, resource allocation between urban and suburban areas, as well as between urban and rural regions, has become increasingly balanced. The location selection outcomes of this study are all situated in transitional zones where urban and rural areas intersect, incorporating weighted considerations related to agricultural product origins, destinations, and distribution centers. This balance extends to the formation of an integrated urban circulation network. By comprehensively evaluating various circulation elements across the entire metropolitan area, a balanced circulation structure is established—prioritizing accessibility under differentiated weighting schemes—laying the foundational conditions for realizing functional differentiation, hierarchical classification, and systemic coordination of the citywide agricultural product wholesale market system in the future.

Harmonization with Community Living Environments: Large-scale agricultural product wholesale markets generate substantial freight demand. To support daytime trading activities, significant volumes of goods are typically unloaded, stored, and transported during nighttime hours, potentially causing adverse effects such as noise pollution, traffic congestion, and waste accumulation in residential neighborhoods. In this study, “urban compatibility” is adopted as a critical evaluation criterion to prevent the introduction of such facilities from disrupting the integrity of urban living environments. Additionally, the potential for landscape resource development is incorporated as an assessment indicator, encouraging these markets to evolve into high-quality urban functional spaces that support leisure, recreation, and positive environmental experiences for local residents.

### The reference value of location selection results

Globally, in the post-harvest stages of food production—including harvesting, storage, and transportation—the rate of food loss in developing countries is significantly higher than in developed countries, exceeding by approximately 40% to 67%. This indicates a negative correlation between food loss rates and the level of agricultural technology and infrastructure development [73].

In addition, in developing countries, there is a compelling need for a robust supply chain governance strategy to regulate the supply chain actors involved in the logistics and distribution system [74]. According to the World Bank’s comparison of the LPI of about 140 countries around the world, Over the past decade, high-income countries have occupied the top positions in the LPI rankings.

**Fig 23:**
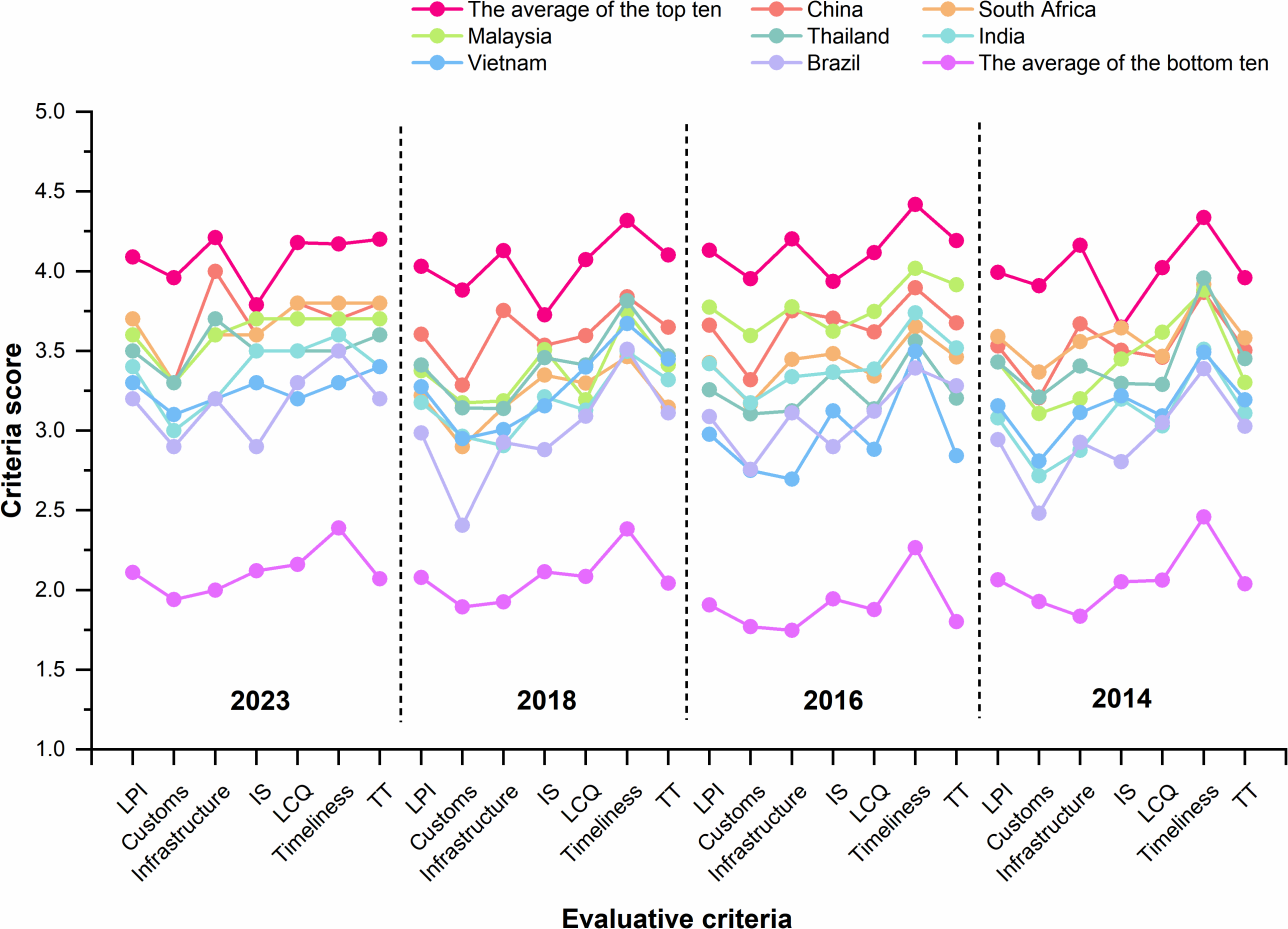
Comparison of LPI Scores among Some Economies. IS, International Shipment; LCQ, Logistics Competence and Quality; TT, Tracking and Tracing. Caption credit: This figure was created by the authors using OriginPro 2024b. The data is sourced from the World Bank’s evaluation reports on the Logistics Performance Index (LPI) of the world’s economies from 2014 to 2022. https://lpi.worldbank.org/international/global.

China’s logistics system has undergone significant improvement in parallel with its economic growth and infrastructure development. As an integral component of this progress, agricultural product logistics has also garnered increased attention and investment. This study analyzes the LPI data [75] for various countries as provided by the World Bank, presenting results for seven developing nations—China, Malaysia, Brazil, India, and others. The findings reveal that between 2014 and 2022, these countries consistently achieved LPI scores above the average of both the top 10 and bottom 10 performers in their respective years, indicating relatively strong logistics performance overall (Fig 23). Given the common trajectories in economic and logistics sector development among developing countries, this analysis may serve as a valuable reference for those currently undergoing rapid modernization of their agricultural product logistics systems.

## Conclusion and future work

The diversity of relevant criteria involved in the location selection for large-scale APWM, as well as the complexity of their relationship with urban and rural areas, constitute the core challenges of location selection. In response to the new demands of modern circulation.

This study examines five major transitions from traditional to modern circulation, summarize existing research findings, and construct an integrated evaluation system for the location selection of large-scale APWM suited for modern circulation. Achieve the integration of territorial and spatial planning, development planning, and urban big data resources in the location selection process. Simultaneously, this approach highlights the integration of a three-dimensional logistics network encompassing land, sea, and air dimensions. A hybrid method combining GIS technology with the AHP and entropy weight methods is proposed, and the comprehensive weights are optimized using GA.

This method, through application in actual case cities, progressively enables regional screening, scope delineation, data analysis, weight optimization, and comprehensive evaluation. It ultimately recommends three alternative plans and conducts a quantitative ranking to identify the optimal solution. Although the ranking and results remained largely unchanged after perturbation and GA optimization, the findings demonstrate that the proposed evaluation system and methodology are effective and stable. Furthermore, it provides scientific support for the location selection of major project planning. This research also serves as a valuable reference and provides guidance for the subsequent development of specialized and detailed plans for territorial space.

In the era of modern circulation, constructing a rational and comprehensive evaluation system and indicator framework to establish a modern agricultural products circulation network space system requires further in-depth research.

In the future, when developing agricultural product wholesale markets or other similar logistics infrastructure systems under the new circulation framework, greater emphasis should be placed on the role of facility location evaluation indicators in promoting social and environmental sustainability. At the same time, the impact of emerging technologies—such as drones, automated cold chain logistics, and e-commerce—must be carefully considered, along with their performance across diverse application scenarios influenced by extreme weather events, diurnal variations, and emergencies such as pandemics. The siting of critical infrastructure essential to maintaining the basic living standards of urban and rural populations should be integrated into regional or municipal emergency preparedness and recovery plans.

**Table:** 

Abbreviation glossary			
**APWM**	Agricultural Products Wholesale Market	**CTSP**	Comprehensive Transportation System Planning of Qingdao (2021–2035)
**PWM**	Production Wholesale Market	**SPFM**	Special Planning for Farmers’ Market of Qingdao (2021–2035)
**DWM**	Distribution Wholesale Market	**SPCN**	Special Planning for Commercial Network of Qingdao (2020–2035)
**CWM**	Consumption Wholesale Market	**14th ARMD**	14th Five-Year Plan for Agricultural and Rural Modernization Development of Qingdao
**ADT**	Average Driving Time	**14th DAPPI**	14th Five-Year Plan for the Development of Agricultural Product Processing Industry in Qingdao City
**NIMBY**	Not-In-My-Back-Yard.	**PLRF**	Proposed List of Recognized Municipal-level Model Family Farms and Farmers’ Cooperatives in Qingdao City for 2023
**AHP**	Analytic Hierarchy Process	**PCMS**	Planning for the Construction of Market System of Qingdao Agricultural Products Producing Area (2015–2020)
**CGCS2000**	China Geodetic Coordinate System 2000	**DEM**	Digital Elevation Model
**POI**	Point of Interest	**TURT**	The Third Phase Construction Plan of Qingdao Urban Rail Transit (2021–2026)
**OD**	Origin and Destination	**GA**	Genetic Algorithm
**TSP**	Territorial Spatial Planning of Qingdao City (2021–2035)	**CV**	Coefficient of variation
**CRTSP**	Corresponding Regional Territorial and Spatial Planning (2021–2035)	**LPI**	Logistics Performance Index
**Nomenclature**			
$w_{a}$	AHP weight	$w_{c}$	Objective weight vector of coefficient of variation
$w_{e}$	entropy weight	$w^{*}$	The comprehensive weight vector optimized by GA
$w_{j}$	The comprehensive weight of normalized linear aggregation	**F(w)**	Weight optimization objective function
$z_{i}$	Comprehensive score	$E_{d}$	Evaluation results of disturbances
$R_{i}$	Contribution rate of weights	$E_{o}$	The original evaluation result
p	Spearman’s rank correlation coefficient	$E_{rate}$	Rate of change in evaluation results

## Supporting information

S1 Table AHP Expert Scoring Table. (XLSX)

S1 Data Publicly available datasets for GIS analysis. (ZIP)

S1 Fig The Third Phase Construction Plan of Qingdao Urban Rail Transit (2021–2026) (Draft for Public Display). (JPG)

S1 Code Genetic Algorithm Code. (ZIP)
