# Supplementary figures and images for: A study of location selection for large agricultural wholesale markets under the perspective of modern circulation
Source: PLoS One. 2026 Apr 7;21(4):e0345727. doi: 10.1371/journal.pone.0345727 (PMC13056211; doi:10.1371/journal.pone.0345727)

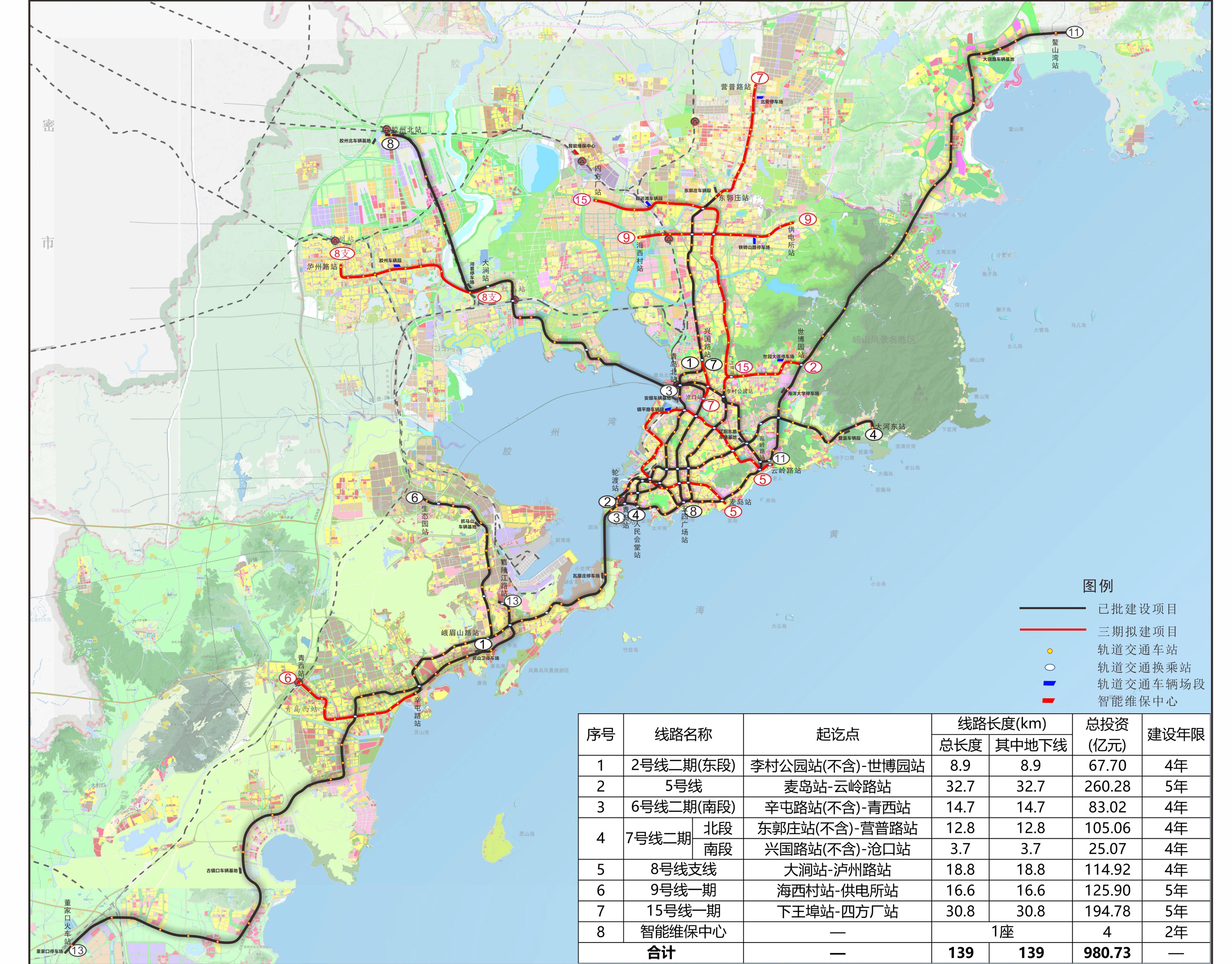

Supplement: S1 Fig — (JPG) [file pone.0345727.s003.jpg]
